# CuI nanoparticle-immobilized on a hybrid material composed of IRMOF-3 and a sulfonamide-based porous organic polymer as an efficient nanocatalyst for one-pot synthesis of 2,4-diaryl-quinolines†

**DOI:** 10.1039/d3ra01164j

**Published:** 2023-04-12

**Authors:** Samaneh Koosha, Sedigheh Alavinia, Ramin Ghorbani-Vaghei

**Affiliations:** a Department of Organic Chemistry, Faculty of Chemistry, Bu-Ali Sina University 6517838683 Hamadan Iran rgvaghei@yahoo.com ghorbani@basu.ac.ir +98-8138380709 +98-8138380709

## Abstract

As a significant class of synthetic and natural products with multiple biological activities, quinolines are used in medical and electronic devices. In this study, a novel method is presented to synthesize 2,4-diarylquinoline derivatives *via* a simple one-pot multicomponent reaction between phenylacetylenes, aniline derivatives, and aldehydes in CH_3_CN using IRMOF-3/PSTA/Cu. Notably, polymer/MOF is stabilized through a reaction between a sulfonamide-triazine-based porous organic polymer [poly (sulfonamide-triazine)](PSTA) and an amino-functionalized zinc metal–organic framework (IRMOF-3). Next, the prepared nanocomposites (IRMOF-3/PSTA) are modified using copper iodide nanoparticles (CuI NPs). Overall, the high product yields, facile recovery of nanocatalysts, short reaction times, and broad substrate range make this process environmentally friendly, practical, and economically justified.

## Abstract 2

As a significant class of synthetic and natural products with multiple biological activities, quinolines are used in medical and electronic devices.

## Graphical abstract

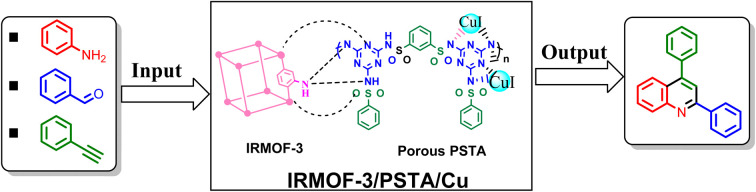

## 1. Introduction

Recently, transition-metal-catalyzed reactions have become an important and powerful procedure in organic synthesis.^1–4^ These reactions follow a more effusive trajectory than the classical ones. Catalysts based on CuI NPs have various organometallic complexes. Therefore, have become very important regarding their efficiency and universal influence on many catalytic reactions.^5–7^ One of the most important reasons for using copper-containing catalysts is their ability to perform carbon–carbon, carbon–nitrogen cross-coupling reactions, and synthesize heterocycle compounds.^8,9^

In addition, metal–organic frameworks (MOFs), as a new category of mesoporous compounds, have numerous found applications in gas storage, catalytic processes, drug delivery, encapsulating materials, supercapacitors, and heavy metal absorbents.^10–13^ MOFs not only have a higher level of activation and stability than other classes of porous materials but also can change the morphology and size of cavities.^14^ Accordingly, this feature has become an advantage in terms of separation and greater selectivity in their applications.^15–17^

MOFs are promising inorganic compounds that can be incorporated into polymer matrices to synthesize polymer/MOF nanocomposites.^18–21^ In this regard, isoreticular organometallic framework-3 (IR-MOF-3) is an important MOF with a cubic framework^22–26^ with a structure containing a 2-aminoterephthalic acid linker and a Zn_4_O cluster.^27^ As a result, this structure has free NH_2_ functional groups not bound to tetragonal ZnO_4_. These features allow IR-MOF-3 to be modified with various organic and organometallic compounds (Fig. 2).^28,29^ Mesoporous polysulfonamides, among others, are compounds of great interest because of their efficiency in preparing heterogeneous catalysts and their use in medicinal chemistry.^30^ Mesoporous polysulfonamides have high chemical stability, high surface area, and low skeletal density.^30,31^ In this sense, a new organic support system consisting of porous sulfonamide triazine-based (PSTA) polymers was prepared using the silica template method (Fig. 2).

MOF/polymer nanocomposites are suitable candidates, functioning as ligands and forming metal complexes, to deposit metal nanoparticles in their substrate regarding their extraordinary features in structural engineering and regular architecture.^32–34^ These compounds have the potential to form complexes because of oxygen groups and nitrogen presence. Therefore, they can be used as excellent support in applications related to catalysis.^10,18,35^ Additionally, the key points in using these nanocomposites as support in heterogeneous catalytic systems are the high specific surface area and easy separation from the reaction mixture.^36^

According to the above-mentioned points, this research aimed at designing and characterizing a novel hybrid crystalline/amorphous material composed of IRMOF-3 and PSTA that offers the advantages of both materials. Moreover, when these agents are combined, it would improve the thermal stability, binding sites, and metal loading levels of the prepared supports. Significantly, the CuI NPs were successfully decorated with the prepared supports (IRMOF-3/PSTA/Cu). Nanocatalysts were successfully incorporated into ternary reactions to prepare new 2,4-diarylquinolines.

**Fig. 1:**
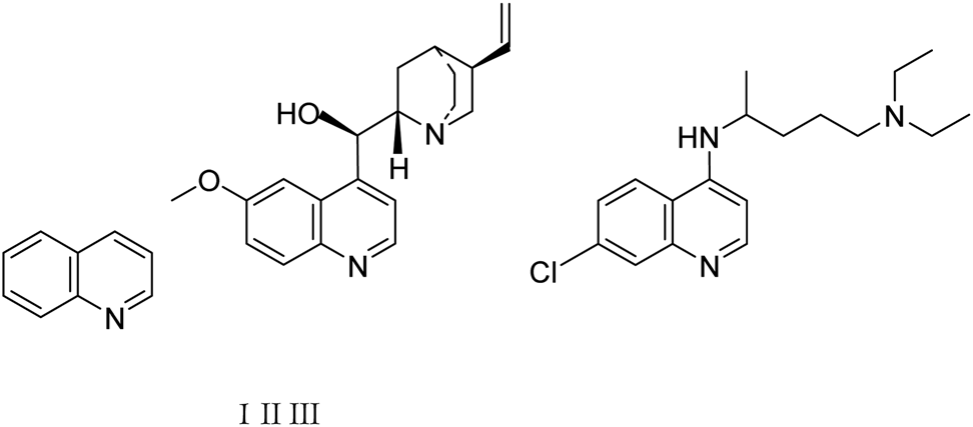
The quinoline (1), the naturally-occurring compound quinine (II), and synthetic chloroquine (III) structures.

**Scheme 1:**
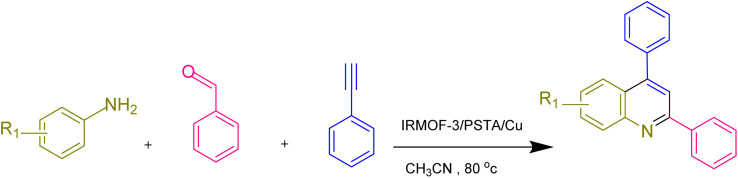
The synthesis of quinoline derivatives using IRMOF-3/PSTA/Cu.

Quinolines are specific heterocyclic pharmacophores with diverse medicinal chemistry and several interesting pharmacological and therapeutic characteristics.^37–39^ They are also used as the core moieties to prepare many well-known drugs with antifungal, antioxidant, and antituberculous activity (Fig. 1).^40–43^ Regarding strategies to synthesize quinoline derivatives, some routes possess specific advantages, *i.e.*, applying safer reagents and solvents, atom economy, being environmentally friendly, and reducing waste production.^44,45^ The method proposed in our work (Scheme 1) is a one-step reaction with an easy purification process and simple recrystallization that takes advantage of the economy of solvents and atoms.

**Fig. 2:**
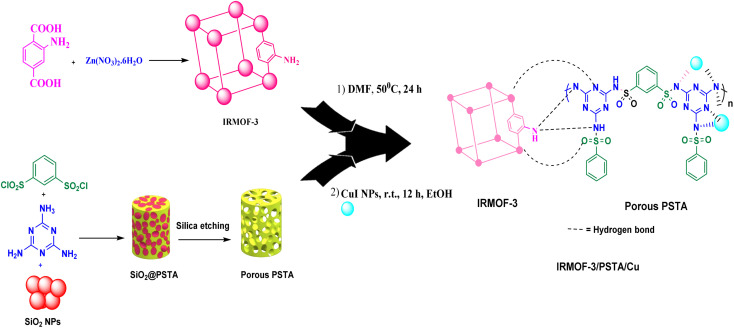
Schematic representation of IRMOF-3/PSTA/Cu.

## 2. Experimental procedures

### 2.1. Materials and methods

Reactions were performed using deionized water. The purchased chemicals (Sigma-Aldrich Chemical Co., St. Louis, MO, USA) were applied without further purification. Thin-layer chromatography was performed on 0.2 mm Merck 60 F254 silica gel aluminum sheets visualized in a UV light chamber equipped with 254 nm and 365 nm lamps followed by applying a Bruker DRX 400 spectrometer for NMR spectra. Moreover, IR spectra (KBr pellets) were recorded by applying a Jasco FTIR model 4600. We also aimed to determine the melting points using an automated STUART melting point SMP50.

### 2.2. Synthesis of IRMOF-3/PSTA/Cu nanocomposites

#### 2.2.1. Synthesis of IRMOF-3

Zn(NO_3_)_2_·6H_2_O (1.895 g) and 2-aminoterephthalic acid (0.370 g) were dissolved in DMF solvent by sonication (50 mL). After ensuring that the container was sealed and impermeable, the solution was heated at 100 °C (in an oven) for 1 day. To remove DMF guest molecules from IRMOF-3, the prepared crystals were isolated, washed with DMF and chloroform three times, and soaked in chloroform for 1 day. Finally, they were dried at 50 °C under reduced pressure^46^ (Fig. 2).

#### 2.2.2. Synthesis of porous PSTA

SiO_2_/PSTA NPs were synthesized through *in situ* polymerization of 1,3,5-triazine-2,4,6-triamine (0.7 mol) and benzene-1,3-sulfonyl chloride (1 mol) in the presence of acetonitrile (30 mL) and SiO_2_ NPs (0.1 g). The reaction mixture was stirred for 6 h under reflux conditions. Afterward, the nanocomposites were washed using ethanol and water. In the next step, it was filtered and vacuum dried at room temperature (RT). Furthermore, the silica template was removed by etching SiO_2_ NPs with an aqueous HF solution. HF solution (10 mL, 10 wt%), deionized water (10 mL), and SiO_2_/PSTA NPs (0.5 g) were added to a 5 mL flask. Then, the mixture was stirred for 6 h at room temperature. Eventually, the produced porous polymer was washed using water and then dried at 50 °C (Fig. 2).

#### 2.2.3. Synthesis of porous IRMOF-3/PSTA

A novel IRMOF-3/PSTA nanocomposite was synthesized by reacting IRMOF-3 with porous PSTA. To this end, 18 mg of IRMOF-3 was dispersed in *N*,*N*-dimethylformamide (5 mL) followed by adding PSTA (12 mg in 5 mL DMF) to the mixture dropwise. Afterward, the mixture was stirred and heated overnight at 50 °C. Finally, the solid was dried at 110 °C for 24 h to remove residual solvent and yield the IRMOF-3/PSTA (Fig. 2).

#### 2.2.4. Synthesis of the IRMOF-3/PSTA/Cu nanocomposite

About 0.1 g of the prepared IRMOF-3/PSTA support was added to an ethanol solution of CuI NPs (0.05 g in 20 mL), followed by stirring the mixture for 12 h. IRMOF-3/PSTA/Cu nanocomposites were then isolated, washed with ethanol (2 × 25 mL) and water (2 × 25 mL), and, dried at 50 °C (Fig. 2).

### 2.3. General procedure to prepare 2,4-diarylquinoline derivatives in the presence of the IRMOF-3/PSTA/Cu catalyst

Phenylacetylene (1.2 mmol), benzaldehyde (1.1 mmol), aniline derivative (1.0 mmol), and IRMOF-3/PSTA/Cu (0.01 g, 0.88 mol%) in CH_3_CN (2 mL) were reacted for 1 hour at 80 °C while stirring. The reaction progress was monitored by applying thin-layer chromatography (TLC). Moreover, centrifugation was used to filter the catalyst, and we also applied ethyl acetate (2 mL) to extract the product (Scheme 1).

## 3. Results and discussion

### 3.1. IRMOF-3/PSTA/Cu nanocomposite characterization

The morphology and structure of the prepared IRMOF-3, SiO_2_/PSTA, porous PSTA, IRMOF-3/PSTA, and IRMOF-3/PSTA/Cu nanocomposites were investigated using FT-IR spectroscopy, energy dispersive X-ray spectroscopy (EDXS), scanning electron microscopy (SEM), thermogravimetric analysis (TGA), Brunauer–Emmett–Teller (BET) analysis, X-ray diffractometry (XRD), and spectral analysis (XPS).

**Fig. 3:**
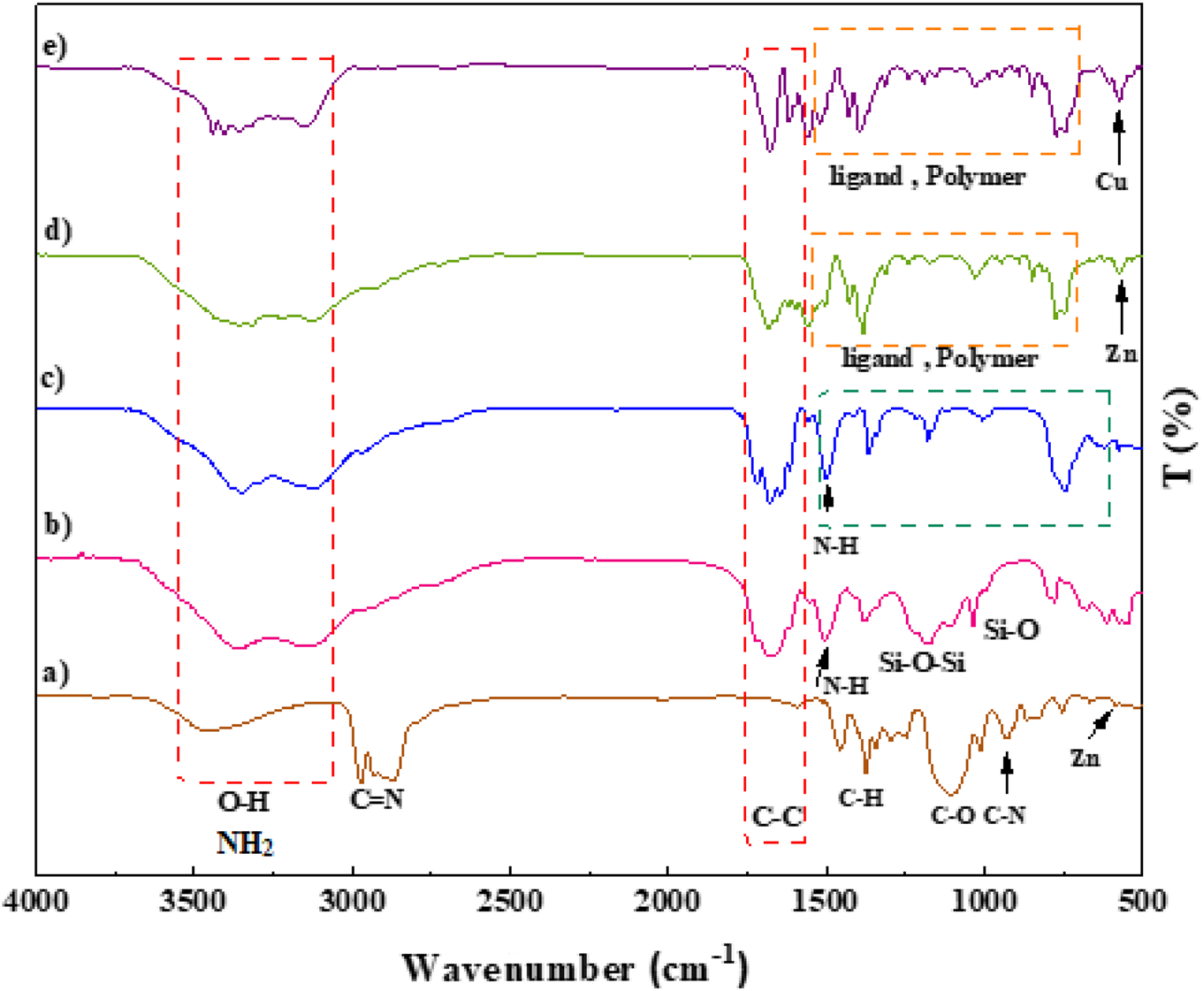
FT-IR spectra of IRMOF-3 (a), SiO_2_/PSTA (b), porous PSTA (c), IRMOF-3/PSTA (d), and IRMOF-3/PSTA/Cu (e).

A prominent peak appeared at 3000–4000 cm^−1^ corresponding to the amino group of the stretching vibration.^47^ The sharp bands from 1682 to 1344 cm^−1^ signified C=C stretching vibrations of a benzene ring and symmetric and asymmetric modes of the dicarboxylate groups.^48^ Furthermore, the peaks at 1101 and 830 cm^−1^ correspond to the aromatic C–H bending vibrations.^49^ The absorption peak at 681 cm^−1^ is due to the metal–oxygen bond of Zn–O (Fig. 3a).^16,50^ Regarding the SiO_2_/PSTA sample, the weak peak at 812 cm^−1^ and the sharp and broad absorption peaks at 1099 cm^−1^ are attributed to symmetric and asymmetric Si–O–Si bond stretches, respectively.^51^ Vibrational bands observed at 1160 and 1338 cm^−1^ represent the presence of a sulfone bridge in the catalyst.^52^ Absorption peaks at 1660, 1680, and 1645 cm^−1^ are related to the melamine presence in the studied structure (Fig. 3b).^53,54^ We did not observe SiO_2_ NPs peak in the FT-IR spectra porous PSTA because of the s selective removal of the template (Fig. 3c). After the hybridization of IRMOF-3 with porous PSTA, the characteristic absorption bands (as previously described) were observed (Fig. 3d). Adding CuI NPs to the structure of IRMOF-3/PSTA significantly changed the FTIR spectra (1685 *vs.* 1665 cm^−1^). Moreover, the bands at 3344 cm^−1^ and 3248 cm^−1^ corresponding to the NH_2_ stretching vibrations shifted to a lower wavenumber compared with 3355 cm^−1^ and 3257 cm^−1^ due to the NH_2_ stretching vibrations of IRMOF-3/PSTA/Cu (Fig. 3e).

**Fig. 4:**
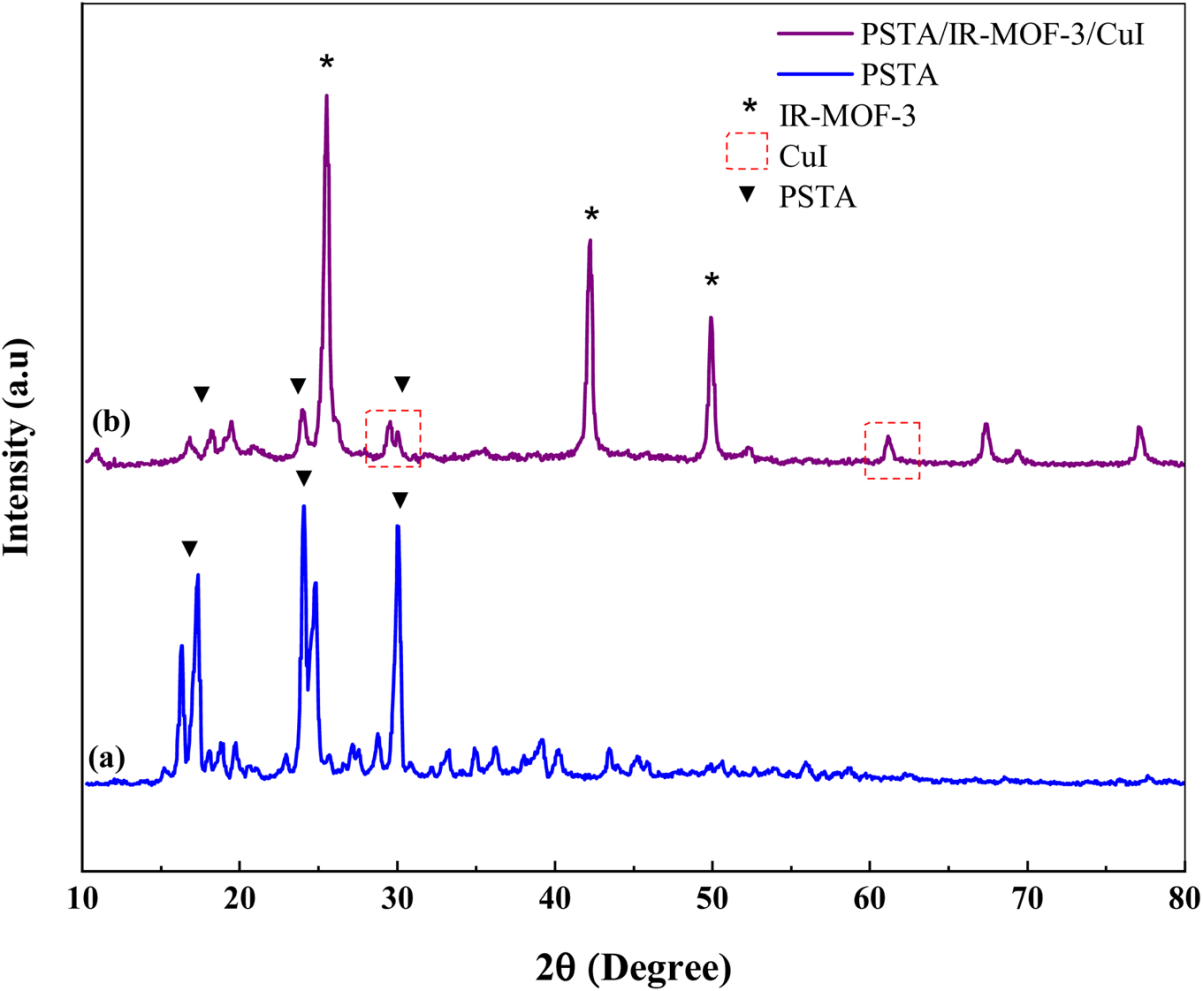
XRD pattern of porous PSTA (a) and IRMOF-3/PSTA/Cu (b).

XRD measurements were performed to evaluate the crystal structures of the prepared porous IRMOF-3/PSTA/Cu and PSTA (Fig. 4). The XRD pattern of the prepared porous PSTA revealed a high degree of crystallization and characteristic peaks of sulfonamides and triazines at 2*θ* = 17.34°, 24.04°, and 29.99° 65. Furthermore, no peaks for SiO_2_ NPs were observed in the XRD pattern of porous PSTA (Fig. 4a). As can be seen from the XRD pattern of the final catalyst (Fig. 4b), the XRD pattern of the synthesized IRMOF-3/PSTA/Cu catalyst has all the characteristic peaks of IRMOF-3 (2*θ* = 25.54°, 42.24°, and 49.89°).^55^ Moreover, porous PSTA (2*θ* = 18.19°, 23.94°, 29.99°) and CuI NPs (2*θ* = 29.54°, 61.29°)^31^ demonstrated successful hybridization and inorganic and organic phases in nanocomposites. Herein, the peak intensity of PSTA decreases at 2*θ* = 18.19°, 23.94° and 29.99°. This change is attributed to the interaction between PSTA and IRMOF-3. Besides, using the Scherer equation, the size of the IRMOF-3/PSTA/Cu nanocomposite crystal was calculated as 3.56 nm, indicating the obtained crystal structure of IR-MOF-3.

**Fig. 5:**
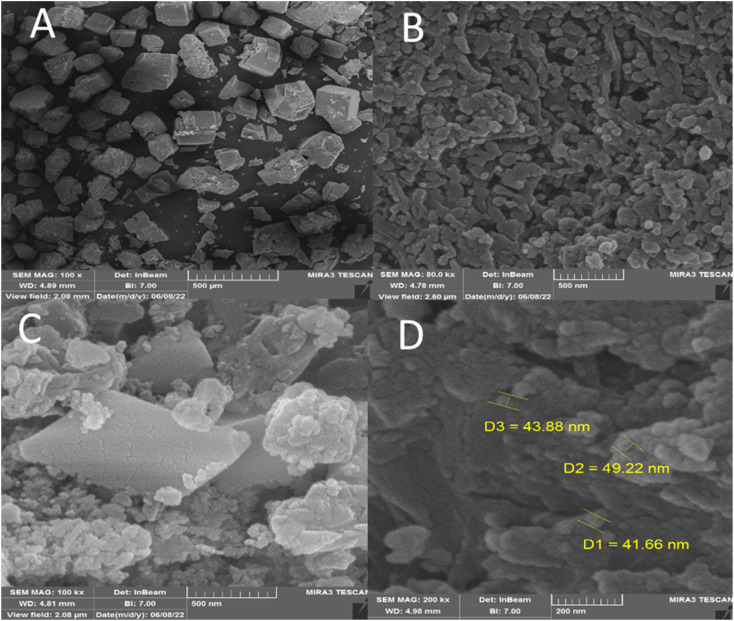
SEM images of IRMOF-3 (A), porous PSTA (B), IRMOF-3/PSTA (C), and IRMOF-3/PSTA/Cu NCs (D).

FESEM was performed to investigate the size and surface morphology of IRMOF-3, porous PSTA, IRMOF-3/PSTA, and IRMOF-3/PSTA/Cu (Fig. 5). A well-distributed, nearly cubic, and uniform nanostructure with a grain size of 75 ± 10 nm was observed in the synthesized IRMOF-3 (Fig. 5A). In addition, FESEM images of PSTA (Fig. 5B) were captured for SiO_2_/PSTA nanocomposites using the selective removal of the silica template. As can be seen, the prepared sample has a 3D porous, regular network with numerous spherical nanopores. The FESEM image of IRMOF-3/PSTA indicates the spherical structure of PSTA developed on IRMOF-3's surface. Moreover, they exhibited a complex and porous nature (Fig. 5C). Notably, the spherical CuI particle distribution on IRMOF-3/PSTA was evident in the FESEM images of the prepared nanocatalysts (Fig. 5D).

**Fig. 6:**
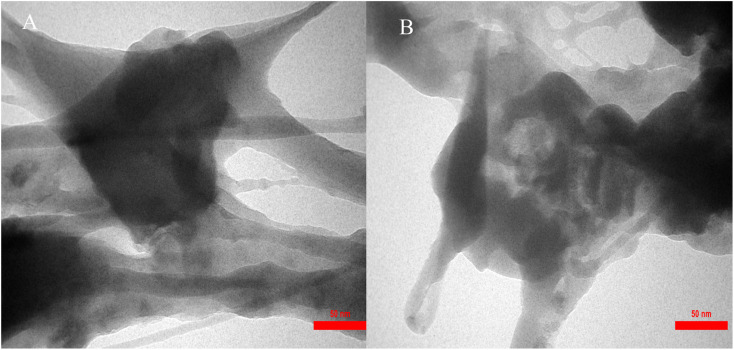
TEM images of IRMOF-3/PSTA/Cu NCs at a scale bare of 50 nm (A) and (B).

The size distribution of IRMOF-3/PSTA/Cu was investigated using transmission electron microscopy (TEM) (Fig. 6). The TEM images of IRMOF-3/PSTA/Cu show a nanocomposite structure.

**Fig. 7:**
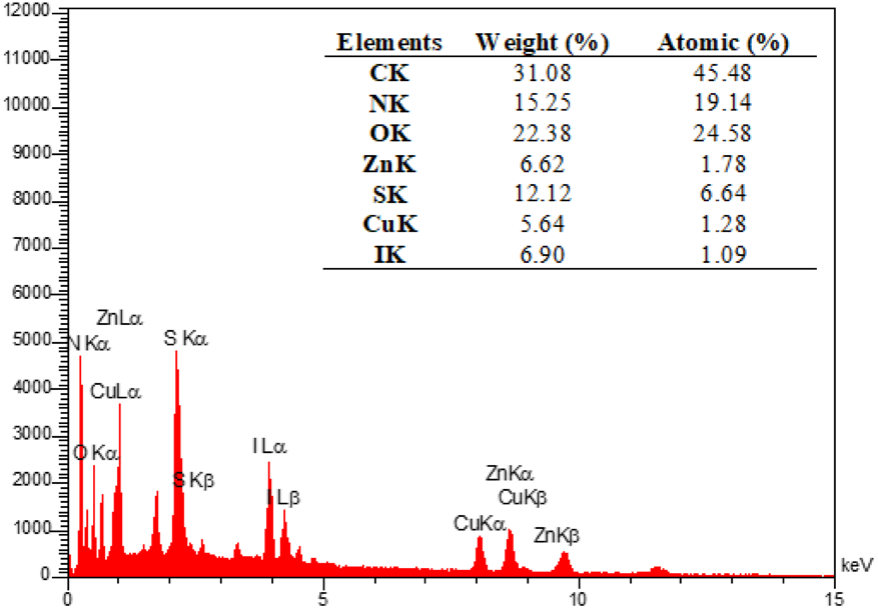
EDX spectrum of IRMOF-3/PSTA@Cu nanocomposite.

**Fig. 8:**
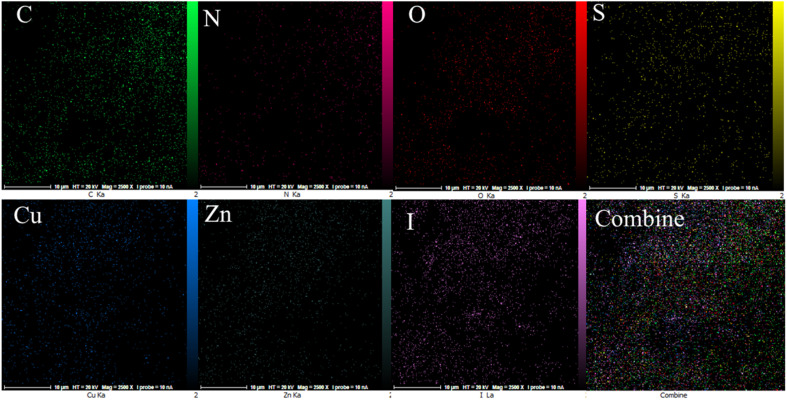
C, N, O, S, Cu, Zn, and I elemental mapping.

The EDX analysis was performed to determine the elemental composition of the IRMOF-3/PSTA/Cu NPs, which confirmed the presence of catalytic components (Fig. 7). Elemental mapping studies of IRMOF-3/PSTA/Cu NPs showed a uniform distribution of carbon, copper, oxygen, iodide, nitrogen, sulfur, and zinc components in the fabricated structure (Fig. 8). In addition, the ICP-AES analysis of IRMOF-3/PSTA/Cu showed 0.88 mmol g^−1^ Cu.

**Fig. 9:**
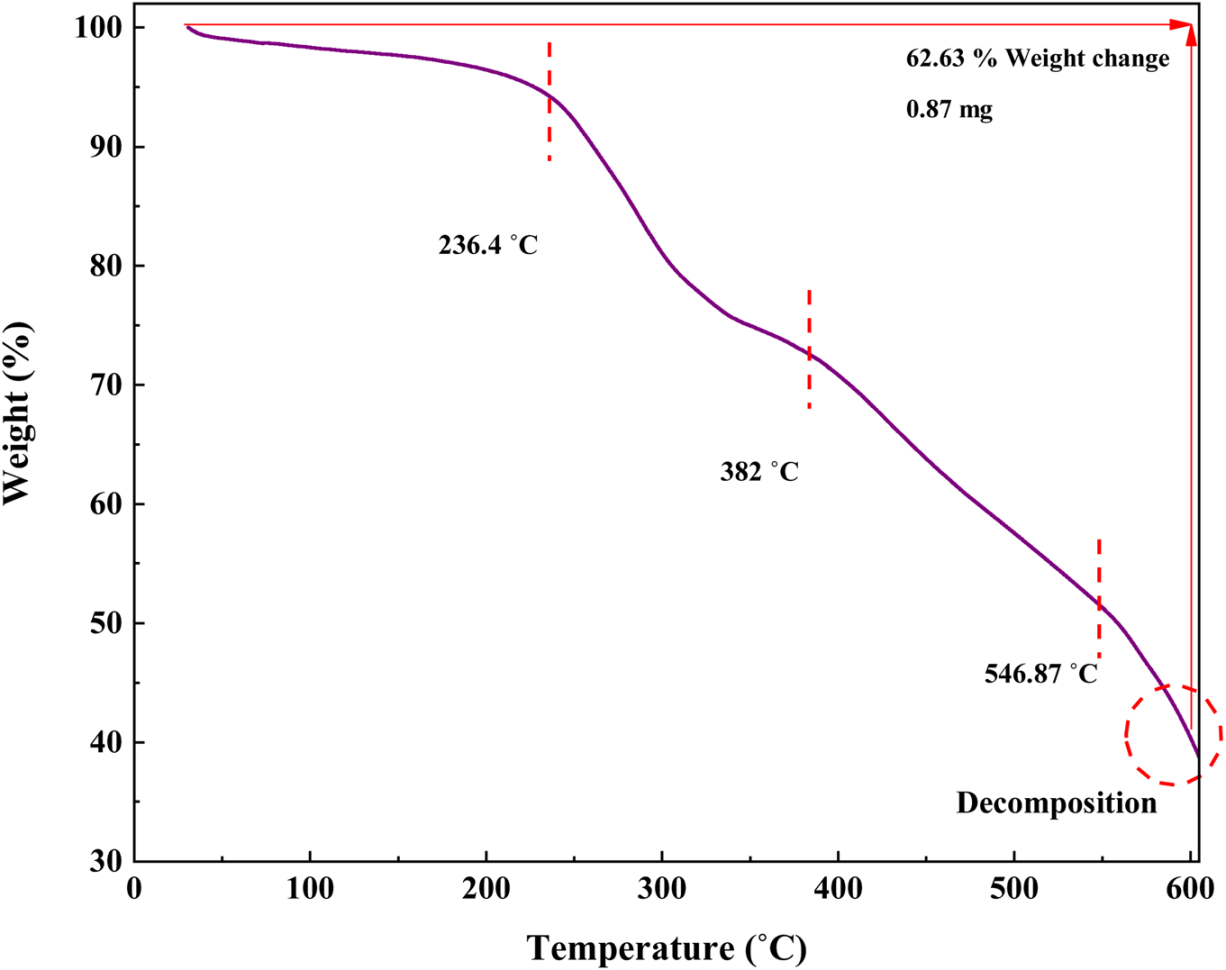
TGA curve of IRMOF-3/PSTA/Cu.

In this research, TGA was performed to investigate the stability of the prepared catalyst (Fig. 9). The TGA curve of IRMOF-3/PSTA/Cu shows three stages of degradation. Through steps 1, 2, and 3, this composite decomposes at 59.39, 382, and 546.87 °C, respectively, typically losing 62.63% of its weight. The first step, starting at 25–236.4 °C, is attributed to solvent removal and thermal condensation of melamine to produce NH_3_ and carbon nitride (CN).^46^ Step 2, starting at 236.4–382 °C, is due to the decomposition of the polymer backbone. Finally, step 3, *i.e.*, from 382 to 546.87, is attributed to the MOF backbone (Fig. 9).

**Fig. 10:**
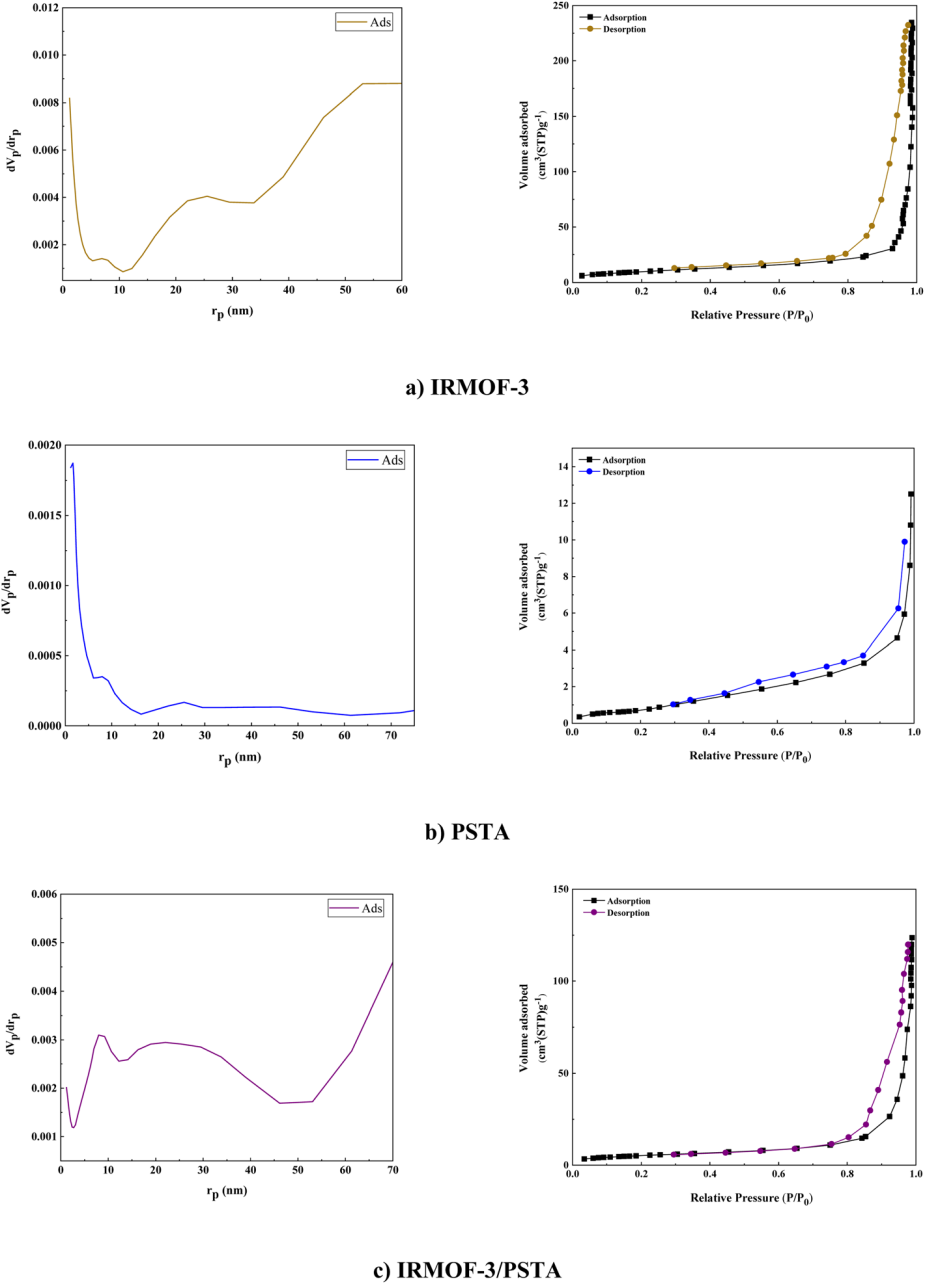
N_2_ adsorption–desorption isotherms of IRMOF-3 (a), PSTA (b), and IRMOF-3/PSTA (c).

BET analysis was carried out on the N_2_ adsorption/desorption isotherms of IRMOF-3, PSTA, and IRMOF-3/PSTA nanostructures. A characteristic type IV H4 hysteresis loop was observed in the synthesized compound, indicating an ordered mesoporous structure (Fig. 10). The specific surface areas of IRMOF-3, porous PSTA, and IRMOF-3/PSTA are 34.96, 2.67, and 19.05 m^2^ g^−1^, respectively (Table 1). As expected, the observed decrease in the synthesized composites is mainly due to the modification of IRMOF-3 by porous PSTA.

**Table 1:** The obtained texture parameters of the materials

Parameter	IRMOF-3/PSTA	PSTA	IRMOF-3
*a*_s_ (m^2^ g^−1^)	34.96	2.67	19.05
*V*_m_ (cm^3^ (STP) g^−1^)	11.31	0.81	6.1
*V*_p_ (cm^3^ g^−1^)	0.36	0.017	0.18
*r*_p_ (nm)	53.04	1.64	71.9
*a*_p_ (m^2^ g^−1^)	34.54	5.64	19.52

**Fig. 11:**
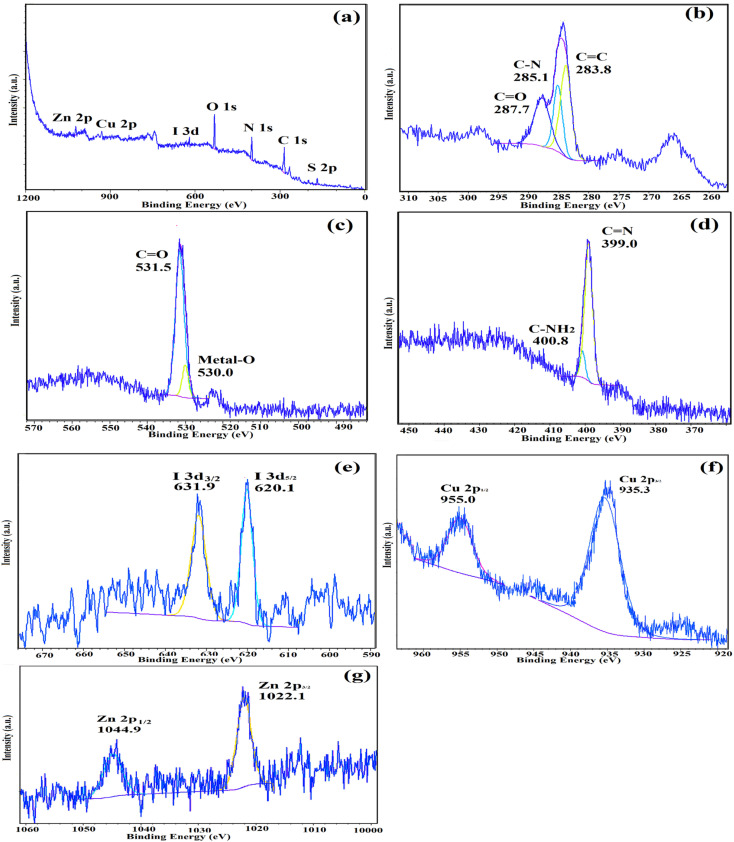
XPS spectra of IRMOF-3/PSTA/Cu: (a) survey, (b) C 1s, (c) O 1s, (d) N 1s, (e) I I 3d, (f) Cu 2p, and (g) Zn 2p.

The atomic oxidation states and chemical compositions of IRMOF-3/PSTA/Cu were investigated using XPS. According to the XPS analysis (Fig. 11a), the basic elements in IRMOF-3/PSTA/Cu are Cu, I, C, O, N, S, and Zn, which is consistent with the EDS analysis results. According to Fig. 11b, the absorption peaks of C1 at binding energies of 287.7 eV, 285.1 eV, and 283.8 eV are assigned to C=C, C–N, and O=C–O, respectively, corresponding to amino terephthalate ligands. Fig. 11c indicates the spectrum of O 1s. Peaks at 530.0 eV and 531.5 eV correspond to M–O (M = Zn, Cu) and O=C–O, respectively. Two peaks at 399.0 eV and 400.8 eV correspond to the functional groups C–NH_2_ and C=N, respectively (Fig. 11d). The observed I 3d peaks in Fig. 11e are characteristic of CuI NPs (Fig. 11e). Moreover, the Cu 2p spectrum indicates two strong peaks at 935.3 eV and 955.0 eV, related to Cu 2p3/2 and Cu 2p1/2 (Fig. 11f), respectively. This observation is a strong reason to prove the interaction of Cu(i) and IRMOF-3/PSTA. The peaks shown in Fig. 11g at 1044.9 eV and 1022.1 eV for Zn 2p in IRMOF-3 are characteristics of Zn 2p1/2 and Zn 2p3/2, respectively. Accordingly, it is confirmed that introducing porous PSTA and CuI NPs into IRMOF-3 resulted in successful surface functionalization.

### 3.2. Model reaction optimization in the presence of (IRMOF-3/PSTA/Cu)

The prepared IRMOF-3/PSTA/Cu was investigated in terms of its catalytic potential and the effects of temperature, catalyst, and solvent content on model reactions. The first optimization test was carried out to obtain the best reaction conditions. The mentioned tests were performed by applying *p*-nitroaniline, benzaldehyde, and phenylacetylene using the IRMOF-3/PSTA/Cu (10 mg). Afterward, the catalyst loading effect was investigated (entries 1–3), with the results shown in Table 2. The optimal MOF level was determined as 10 mg (entry 2), yielding the lowest catalyst consumption and the highest amount of product (*i.e.*, 0.43 mol% Cu for IRMOF-3/PSTA/Cu). The complete transformation of the reactants occurred (based on yield) by adding 10 mg of the catalyst. However, the reaction efficiency was not improved by increasing the amount of IRMOF-3/PSTA/Cu beyond 10 mg. Using ethanol, DMF, and chloroform (entries 5–7), no conversion was observed. It is worth mentioning that the reaction efficiency was decreased using ethanol. Ethanol's deactivating effect is also reflected through changes in the activation energy. Moreover, the decreased activity of the catalytic species is suggested to be caused by preferential solvation and reaction by ethanol. In the case of toluene, the low solubility of the synthesized catalyst in toluene as a solvent aids the reaction with a lower rate and efficiency. Toluene preceded the reaction in low yield (entry 4). This study showed that IRMOF-3/PSTA/Cu has high dispersity in acetonitrile. Hence, CH_3_CN proved to be the best for this reaction, giving 90% product after 1 hour (Table 1, entry 2). Nevertheless, IRMOF-3, UiO-66-NH_2_, Zn(NO_3_)_2_·6H_2_O, CuI NP, IRMOF-3/PSTA, PSTA/CuI, and IRMOF-3/CuI (Table 2, entries 8–14) did not lead to satisfactory results. In addition to the effective metal-support synergistic interaction of copper and IRMOF-3/PSTA in the IRMOF-3/PSTA/CuI catalyst, the Lewis acidic nature of IRMOF-3 immensely contributed to bringing the N-bearing heterocyclic substrate closer to the catalytic metal center (Cu), thereby promoting the reaction rate. Introducing metal nanoparticles into these structures leads to the creation of bimetallic systems. As a result, it brings the synergistic effect of the metal nanoparticles introduced with the metal nodes in the MOF structure and enhances the catalytic activity. Also, the IRMOF-3/PSTA support may promote copper metal particle dispersion on its surface, resulting in more active catalyst sites of the metal center. The evaluation of different temperatures confirmed that 80 °C is a favorable temperature for the model reactions (entry 15). After the screening, optimal results were obtained in the presence of 10 mg of IRMOF-3/PSTA/Cu nanocatalyst with CH_3_CN as the solvent at 80 °C.

**Table 2:** Model reaction optimization^a^

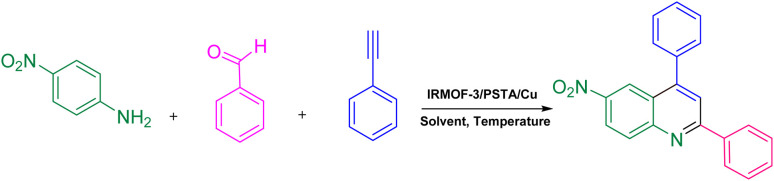					
Entry	Cat. (mg)	Solvent	Temperature (°C)	Time (h)	Yield^b^ (%)
1	IRMOF-3/PSTA/Cu (5)	CH_3_CN	80	1	81
2	IRMOF-3/PSTA/Cu (10)	CH_3_CN	80	1	90
3	IRMOF-3/PSTA/Cu (20)	CH_3_CN	80	1	90
4	IRMOF-3/PSTA/Cu (10)	Toluene	80	1	62
5	IRMOF-3/PSTA/Cu (10)	EtOH	80	1	23
6	IRMOF-3/PSTA/Cu (10)	DMF	80	1	43
7	IRMOF-3/PSTA/Cu (10)	CH_3_Cl_3_	80	1	29
8	IRMOF-3 (10)	CH_3_CN	80	1	55
9	UiO-66-NH_2_ (10)	CH_3_CN	80	1	35
10	Zn(NO_3_)_2_·6H_2_O (10)	CH_3_CN	80	1	Trace
11	CuI NPs (10)	CH_3_CN	80	1	51
12	IRMOF-3/PSTA (10)	CH_3_CN	80	1	60
13	PSTA/Cu (10)	CH_3_CN	80	1	66
14	IRMOF-3/Cu (10)	CH_3_CN	80	1	80
15	IRMOF-3/PSTA/Cu (10)	CH_3_CN	60	1	73

a Reaction condition: *p*-anisidine (1.0 mmol), phenylacetylene (1.2 mmol), benzaldehyde (1.1 mmol), in CH_3_CN (2 mL).

b Isolated yields.

The generality and range of reactions were investigated for various anilines under optimal conditions (Table 3). Based on the obtained results, aniline's electron-withdrawing groups enhanced the IRMOF-3/PSTA supported Cu-catalyzed domino reactions with higher reactivity than those containing electron-donating groups, leading to higher yields (4b*vs.*4e and 4h). The Diels–Alder reaction is the so-called inverse electron claim when the dienophile has an electron-withdrawing group, and the diene possesses an electron-donating group. The reaction with the methyl group of aniline (4i) gave similar yields as with the methoxy group (4b). A comparison of these results with the nitro group (4h) and bromide and chloride groups (4a, 4c, 4e) shows that the yield and reaction rate are functions of the EWG strength. The explanation is that the bromo group is less electron-donating than the nitro group, resulting in lower yields. In this study, we prepared new quinoline derivatives, which are the first to be reported.

**Table 3:** Preparation of quinoline derivatives using IRMOF-3/PSTA/Cu nanocatalyst^a^

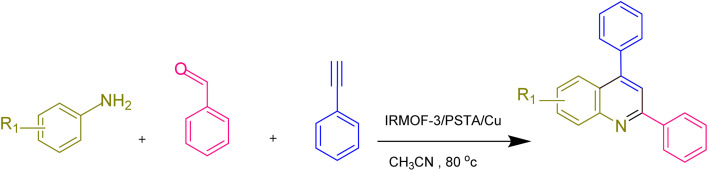						
Entry	Aniline	Product	Time (min)	Yield^b^ (%)	Melting point	
					Measured	Reported (ref.)
1	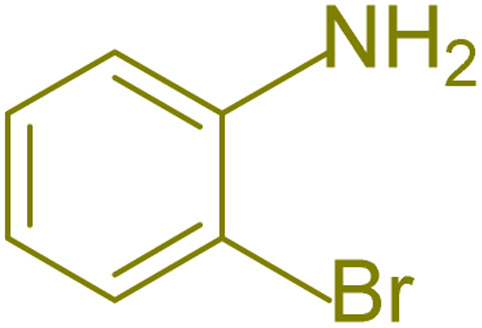	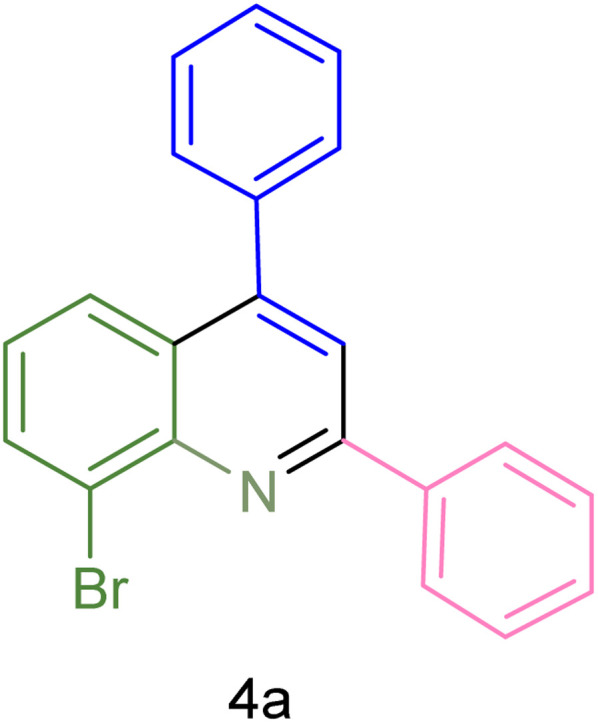	90	82	162–164	New
2	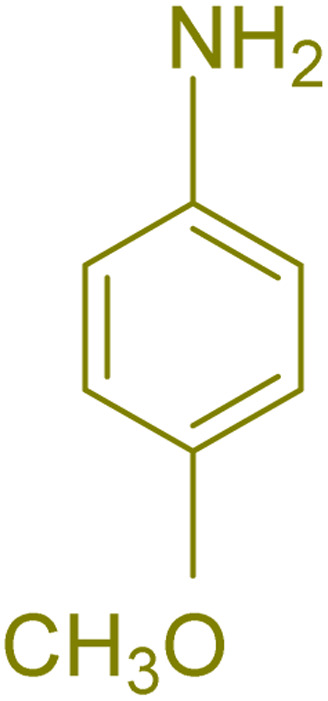	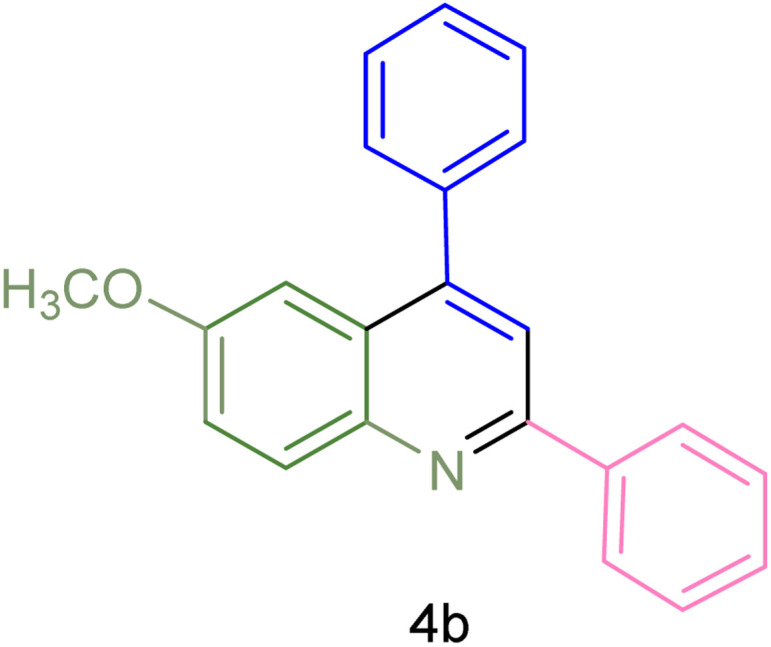	180	76	119–120	121–122 (ref. 56)
3	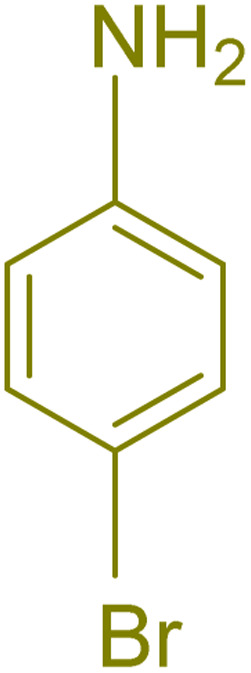	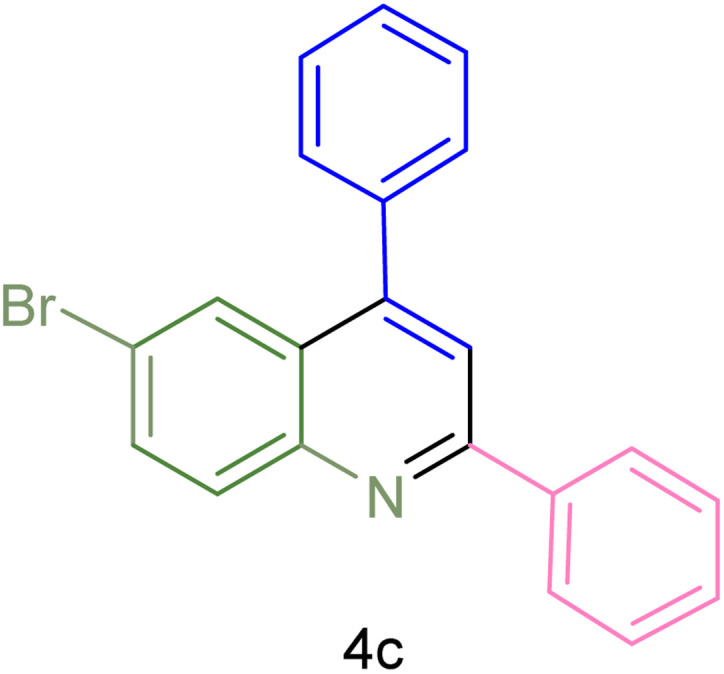	85	84	147–149	150–152 (ref. 57)
4	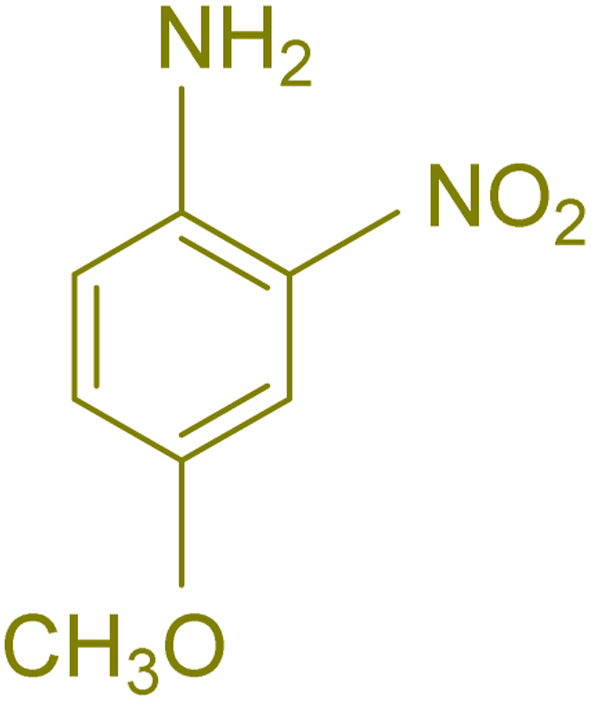	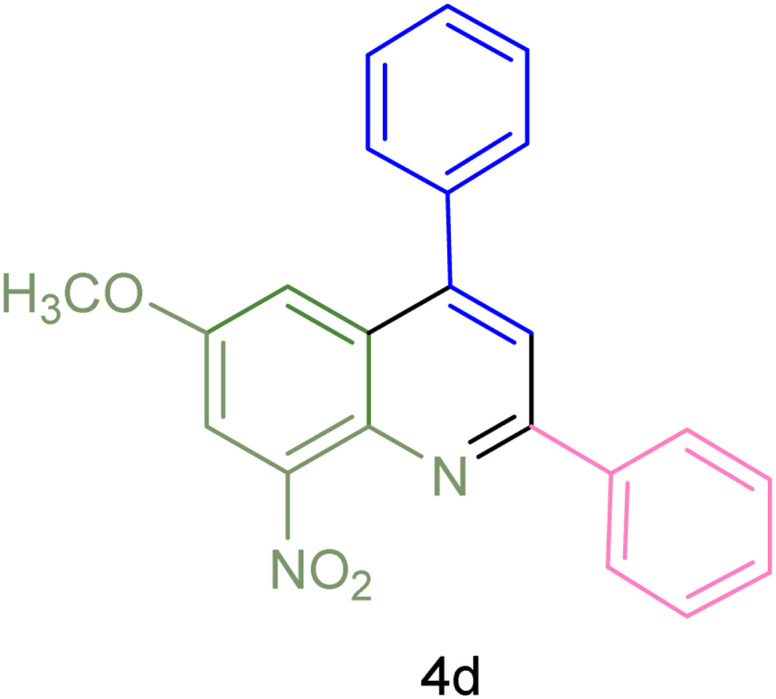	75	85	155–157	New
5	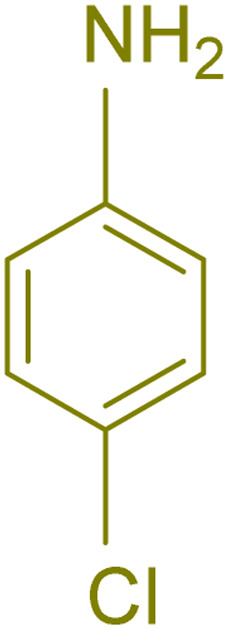	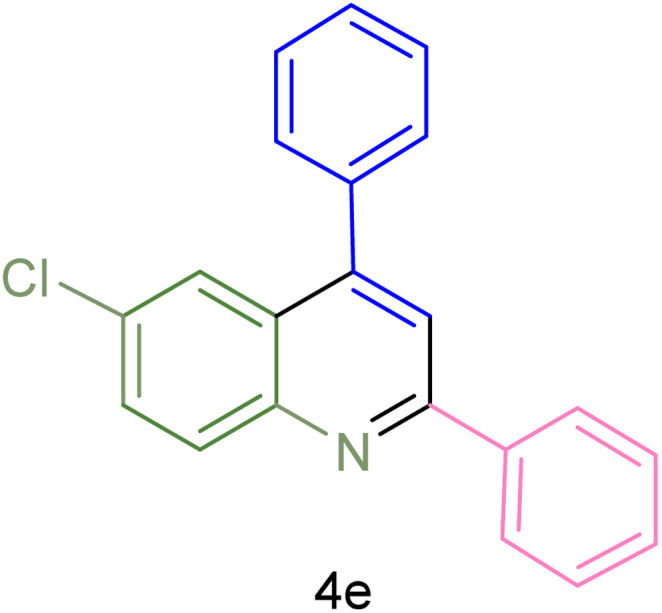	85	80	134–136	131–132 (ref. 58)
6	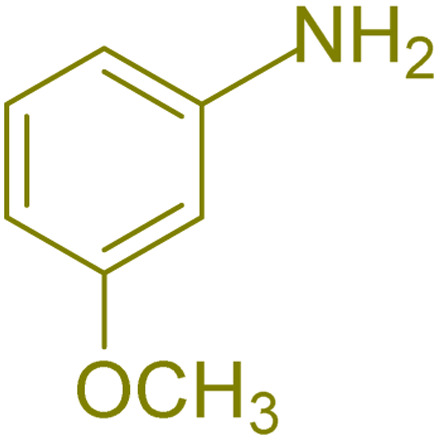	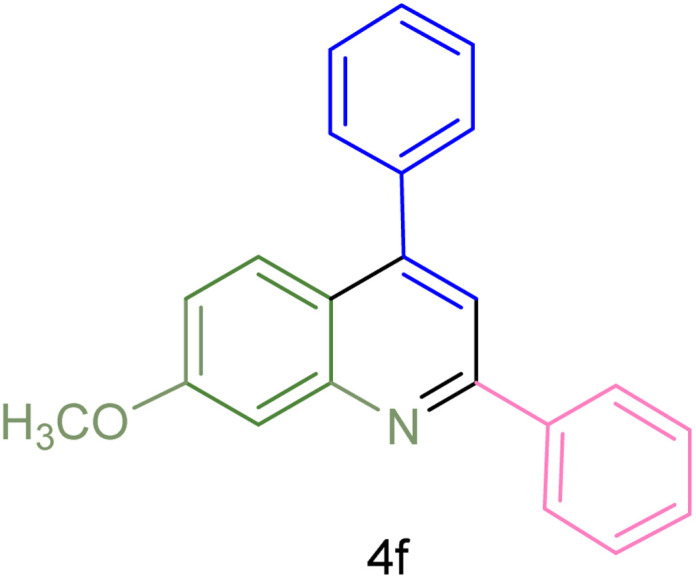	240	74	80–82	81–82 (ref. 59)
7	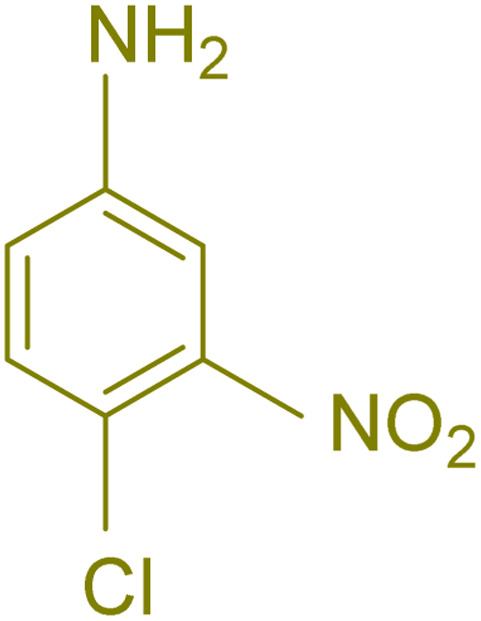	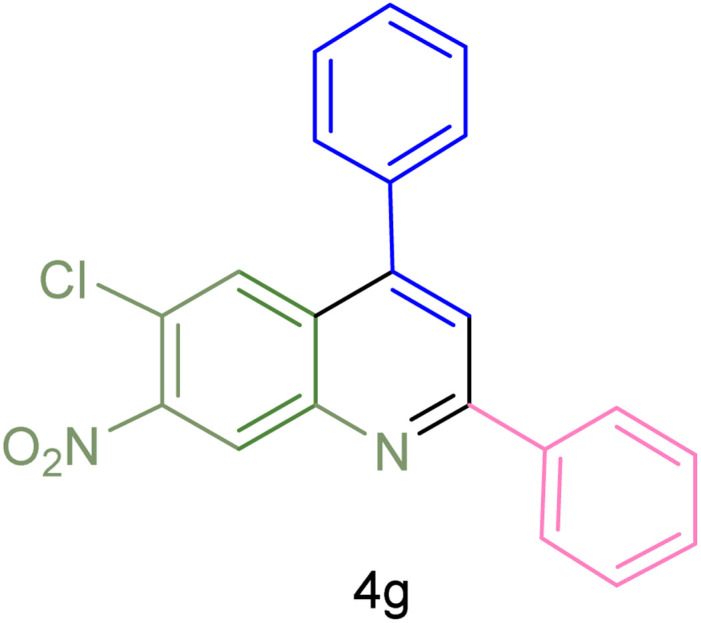	75	83	251–253	New
8	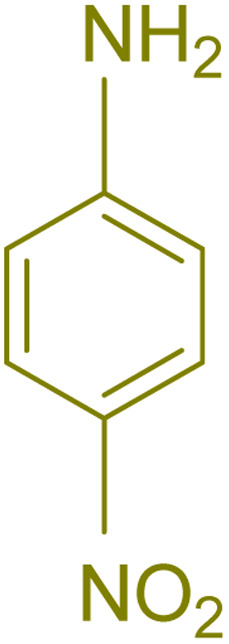	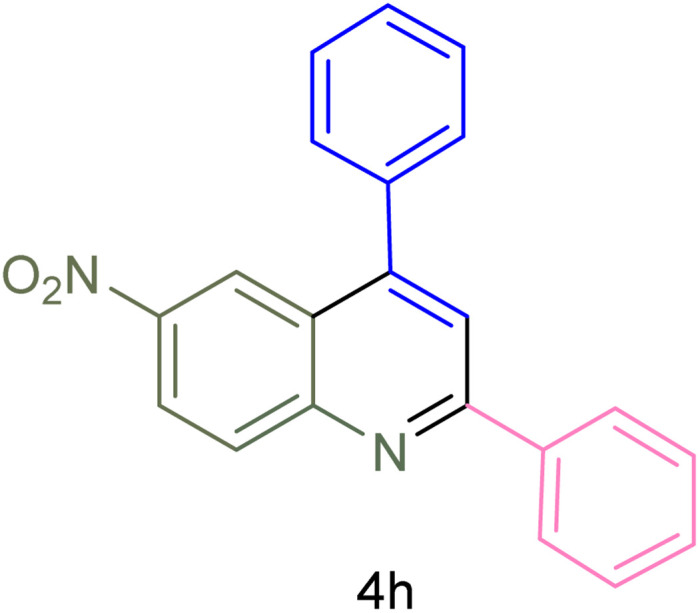	60	90	262–263	265–266 (ref. 60)
9	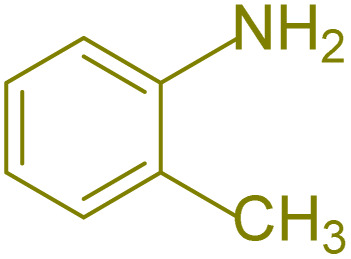	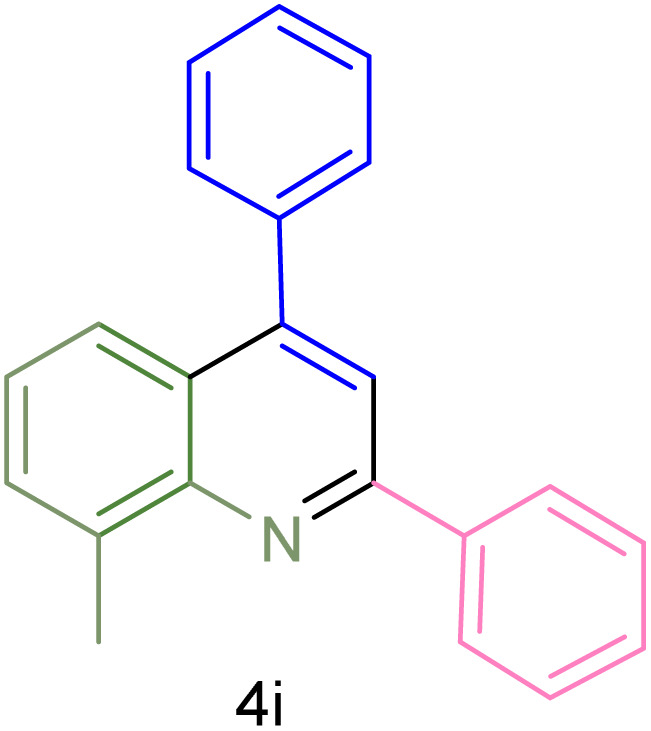	180	78	120–122	122–123 (ref. 61)
10	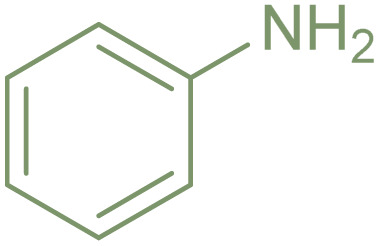	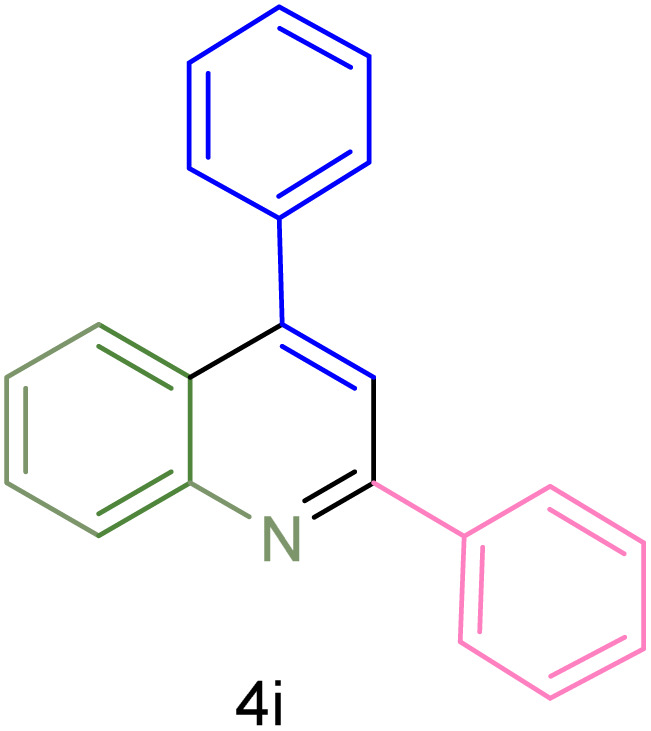	120	88	150–152	150–152 (ref. 56)

a Reaction condition: phenyl acetylene (1.2 mmol), IRMOF-3/PSTA/Cu (0.01 g, 0.88 mol%), aniline derivatives (1.0 mmol), and benzaldehyde (1.1 mmol) in CH_3_CN (2 mL) was heated at 80 °C.

b Isolated yield.

The advantages of the produced nanocatalysts are summarized in Table 4. The table compares the properties of various catalytic systems used to produce 2,4-diarylquinolines in the presence of different catalysts. According to these data, the catalytic system presented in this study offers higher efficiency than other systems for this reaction regarding the reaction yield and time. The advantages of IRMOF-3/PSTA/Cu as the catalyst in the system include a less intensive process, a high yield of 92%, and separability and recyclability up to several times, which are difficult to obtain using pure CuI NPs (entry 4).

**Table 4:** A comparison of our methodology with other ones used to synthesize 2,4-diaryl-quinoline

Entry	Catalyst	Solvent	Conditions	Time (h)	Yield^a^ (%)	Ref.
1	NbCl_5_ (0.5 eq)	CH_3_CN	r.t.	96	36–98	62
2	K_5_CoW_12_O_40_·3H_2_O	—	90	10 min	87–98	60
3	MOF-5	Solvent-free	110	3	87–91	63
4	IRMOF-3/PSTA/Cu	CH_3_CN	90	1	74–90	This work

a Isolated yield.

**Scheme 2:**
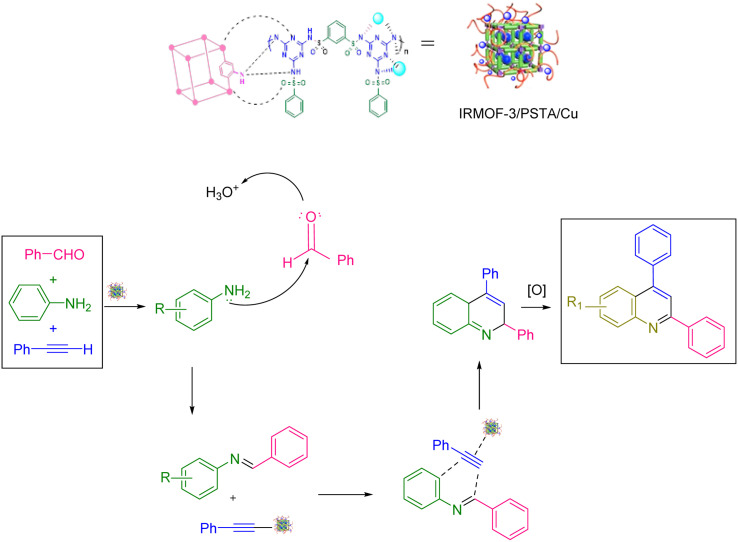
The suggested mechanism.

Scheme 2 shows the mechanism offered for the one-pot preparation of quinoline derivatives catalyzed by IRMOF-3/PSTA/Cu. According to this mechanism, the IRMOF-3/PSTA/Cu catalyst first enhances the activity of phenylacetylene binding to iminium ions generated *in situ* by the reaction between benzaldehydes and amines. Afterward, phenylacetylene reacts with a Schiff base through a concerted cyclization, *i.e.*, the Diels–Alder reaction, to give the dihydroquinoline. Finally, the target products are formed *via* oxidation (Scheme 2).^64^

**Fig. 12:**
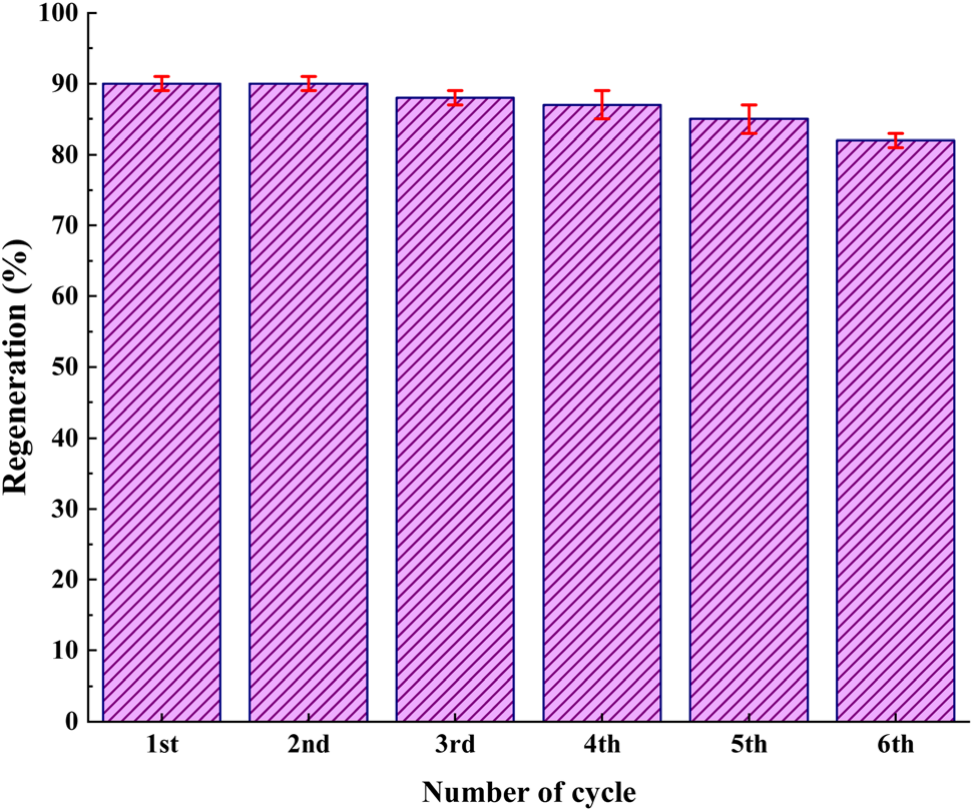
Reusability of IRMOF-3/PSTA/Cu in the synthesis of compound 4h.

**Fig. 13:**
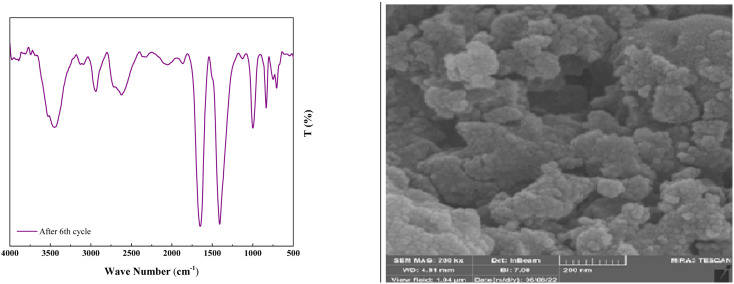
The FT-IR spectrum and FESEM image of the reused catalyst after six recycles.

As shown in Fig. 12, the stability of the catalysts was proved *via* the characterization by FT-IR and FESEM (90, 90, 88, 87, 85, and 82). The outcomes confirmed the stability of the catalysts as the regeneration data do not exhibit any change after the initial patterns (Fig. 13).

## 4. Conclusion

In conclusion, it was feasible to develop a method to synthesize quinoline derivatives in a one-pot multicomponent reaction between phenylacetylene, aniline derivatives, and benzaldehyde using IRMOF-3/PSTA/Cu nanocomposites. This method provides good yields and suitable reaction times. The IRMOF-3/PSTA/Cu nanocomposite was synthesized by reacting IRMOF-3 with porous PSTA, followed by immobilizing CuI NPs. Considering the catalytic activity, high porosity, and large surface area are of great significance. Moreover, IRMOF-3/PSTA/Cu was found to be a multifunctional MOF having stability and robustness. Notably, up to six times, the product could be used without losing structural integrity and catalytic activity. Furthermore, there are also some specific benefits (regarding the suggested mesoporous catalyst), including a clean reaction profile, high yield, and the catalyst's facile separation.

## Conflicts of interest

There are no conflicts to declare.

## Supplementary Material

RA-013-D3RA01164J-s001
