# Supplementary material for: CuI nanoparticle-immobilized on a hybrid material composed of IRMOF-3 and a sulfonamide-based porous organic polymer as an efficient nanocatalyst for one-pot synthesis of 2,4-diaryl-quinolines
Source: RSC Adv. 2023 Apr 12;13(17):11480–94. doi: 10.1039/d3ra01164j (PMC10091365; doi:10.1039/d3ra01164j)
Supplement: RA-013-D3RA01164J-s001 [file RA-013-D3RA01164J-s001.pdf]

Electronic Supplementary Material (ESI) for RSC Advances.
This journal is © The Royal Society of Chemistry 2023

CuI nanoparticles-immobilized on a hybrid material composed of IRMOF-3 and a sulfonamide-based porous organic polymer as an efficient nanocatalyst for one-pot synthesis of 2,4-diaryl-quinolines

Samaneh Koosha, Sedigheh Alavinia, Ramin Ghorbani-Vaghei,*

Department of Organic Chemistry, Faculty of Chemistry, Bu-Ali Sina University, 6517838683, Hamadan, Iran

Corresponding Author: Fax: Tel./Fax: +98-8138380709

E-mail: rgvaghei@yahoo.com & ghorbani@basu.ac.ir

^{1}H NMR (500 MHz, DMSO-d_6, TMS) δ 8.55 (d, *J* = 6.8 Hz, 1H), 8.52 (d, *J* = 3.4 Hz, 2H), 8.09 (d, *J* = 8.6 Hz, 1H), 7.97 (t, *J* = 9.0 Hz, 3H), 7.91 (d, *J* = 7.8 Hz, 1H), 7.60 (d, *J* = 9.1 Hz, 1H), 7.55-7.46 (m, 3H), 7.29 – 7.24 (m, 1H), 6.89 (s, 1H), 3.71(s, 3H). ^{13}C NMR (126 MHz, DMSO-d_6, TMS) δ 163.02, 155.62, 146.68, 145.61, 139.27, 134.78, 134.27, 132.67, 132.18, 127.95, 126.11, 125.05, 115.79, 61.46.

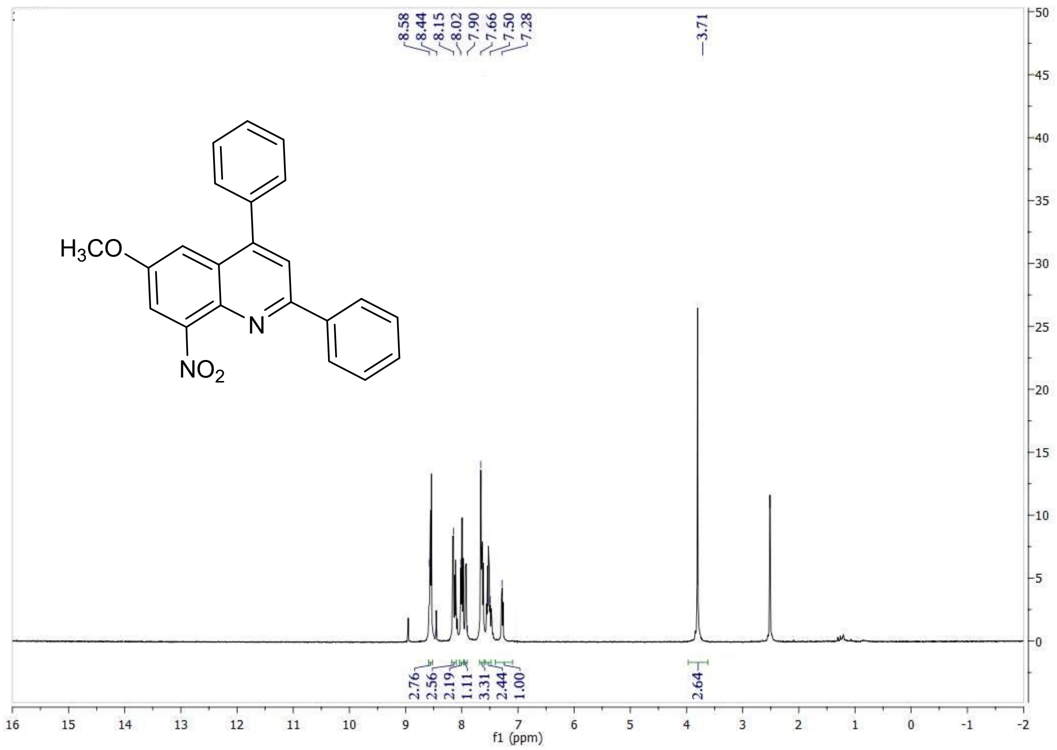

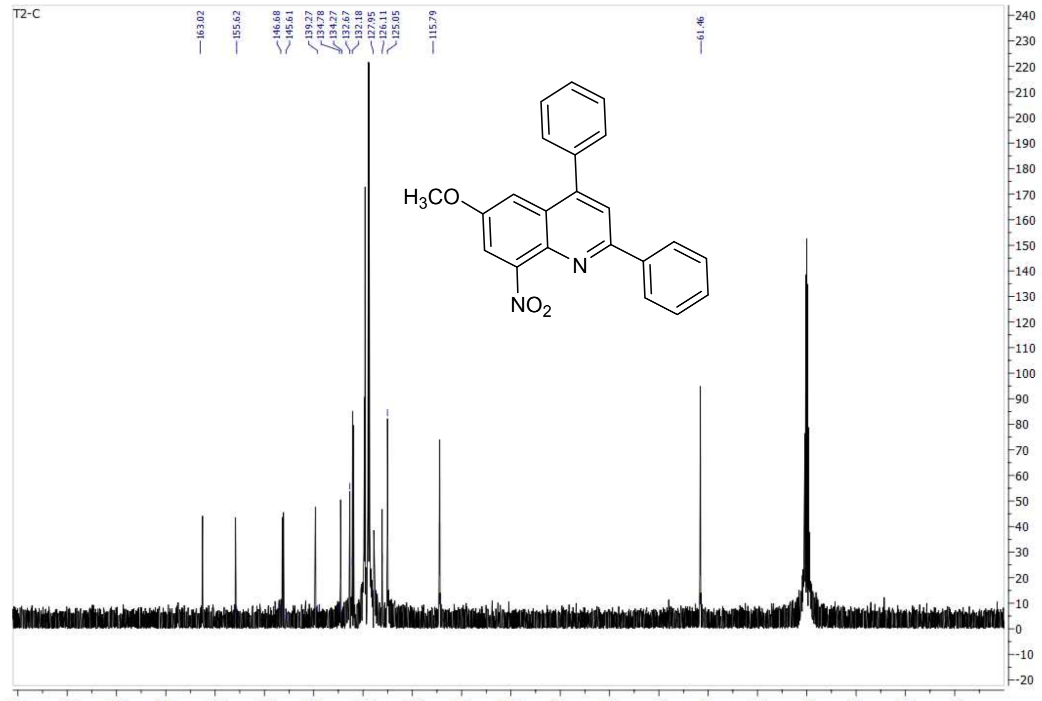

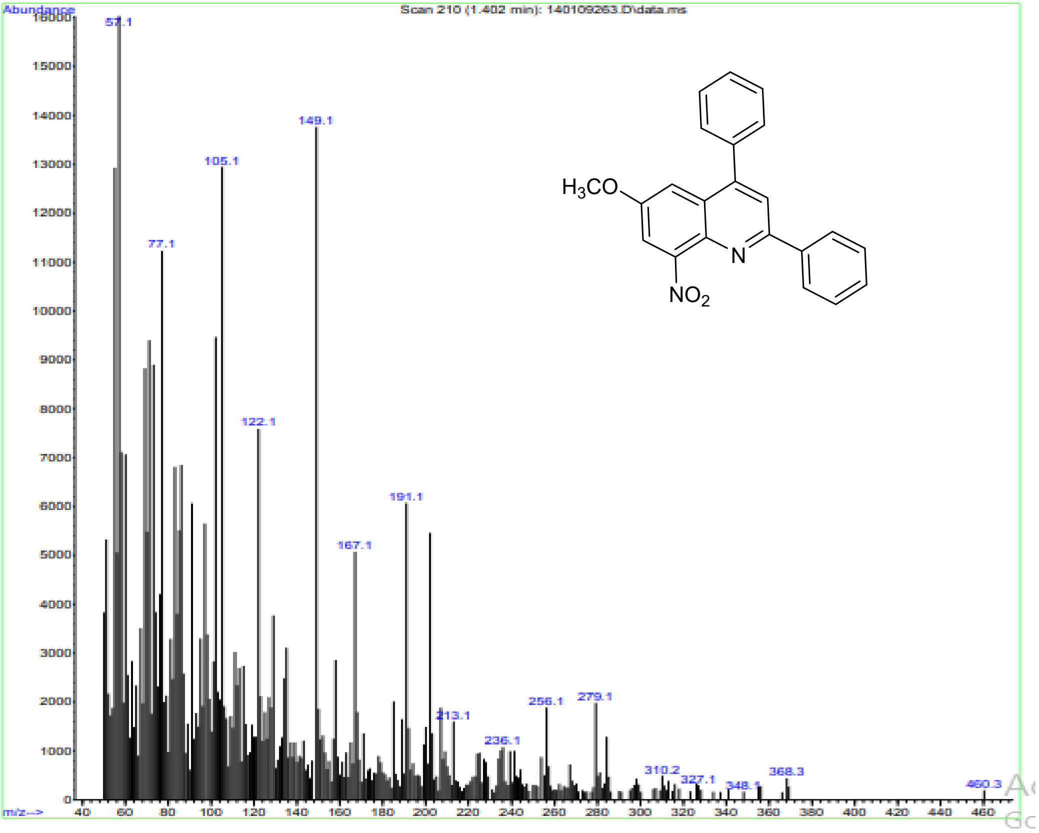

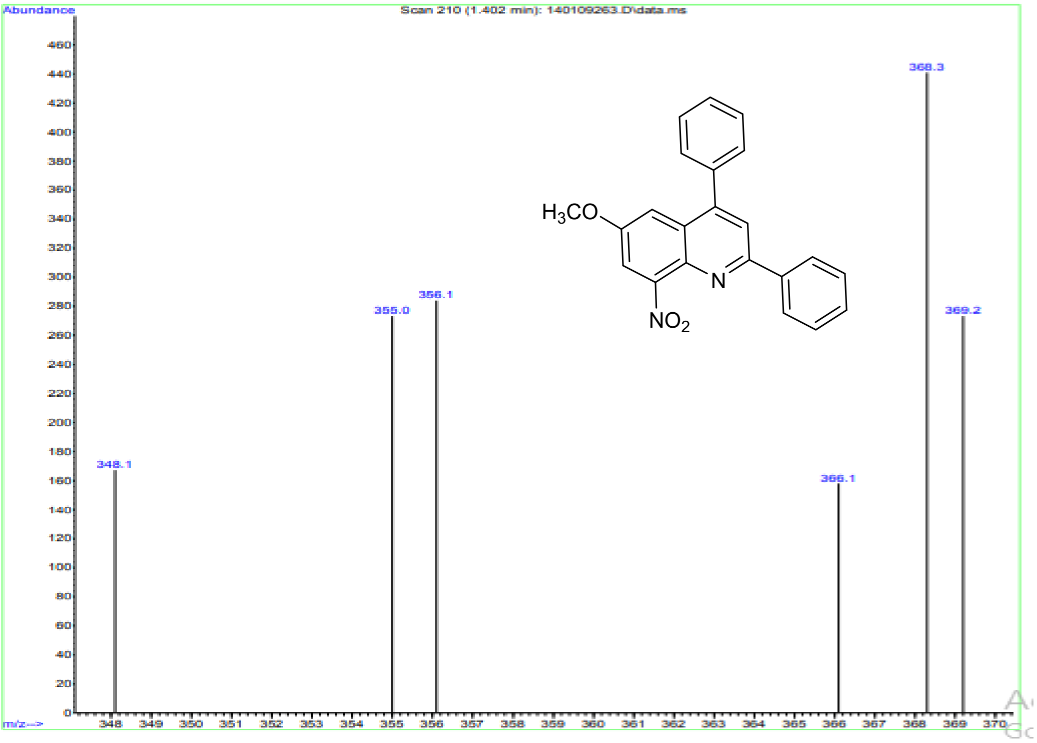

^{1}H NMR (500 MHz, DMSO-d_6, TMS) δ 8.58 – 8.51 (m, 5H), 8.15 (d, J = 7.9 Hz, 3H), 8.12 (d, J = 8.5 Hz, 2H), 8.00 (t, J = 9.7 Hz, 4H), 7.93 (d, J = 7.7 Hz, 2H), 7.68 – 7.62 (m, 5H), 7.62 (s, 1H), 7.56 – 7.49 (m, 4H), 7.35 – 7.19 (m, 2H), 6.91 (s, 2H). ^{13}C NMR (126 MHz, DMSO-d_6, TMS) δ 159.91,

149.43, 133.72, 133.07, 131.93, 128.59, 128.49, 128.05, 127.40, 126.81, 126.34, 125.51, 124.48, 124.28, 117.10, 112.74, 110.15. MS m/z (%): 315.08.

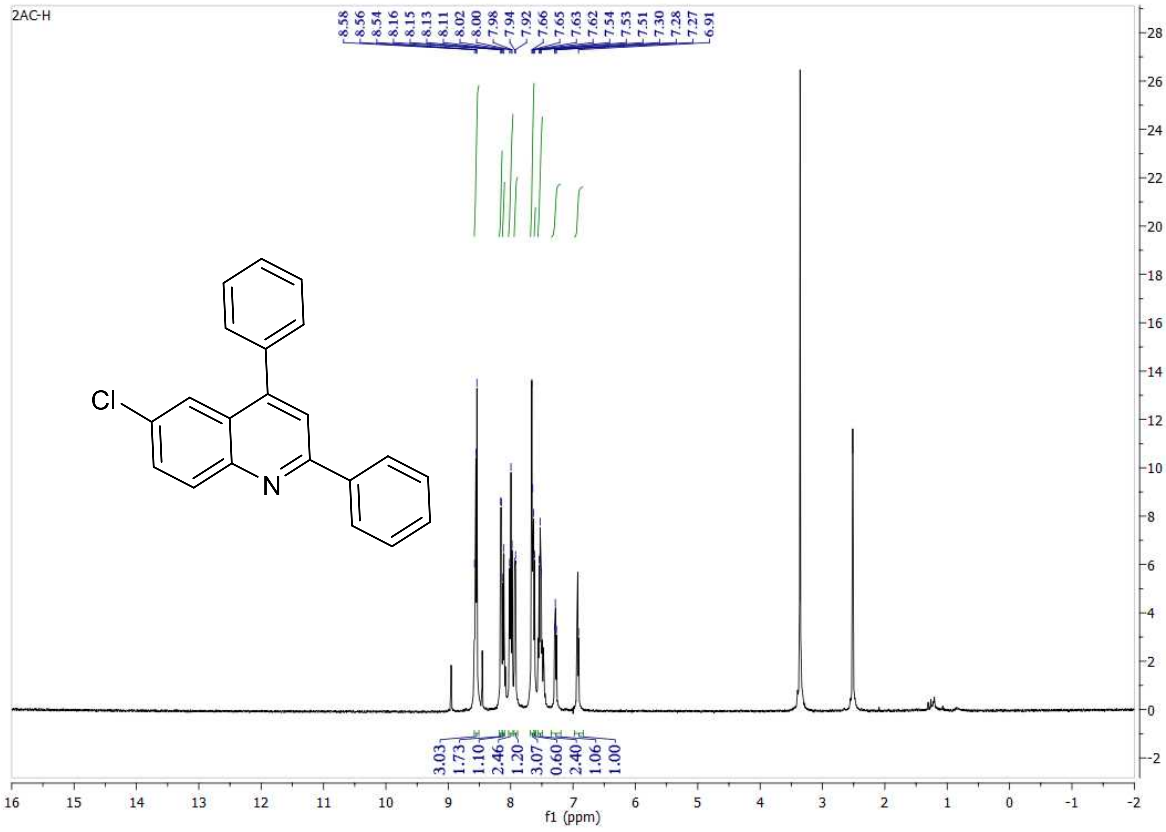

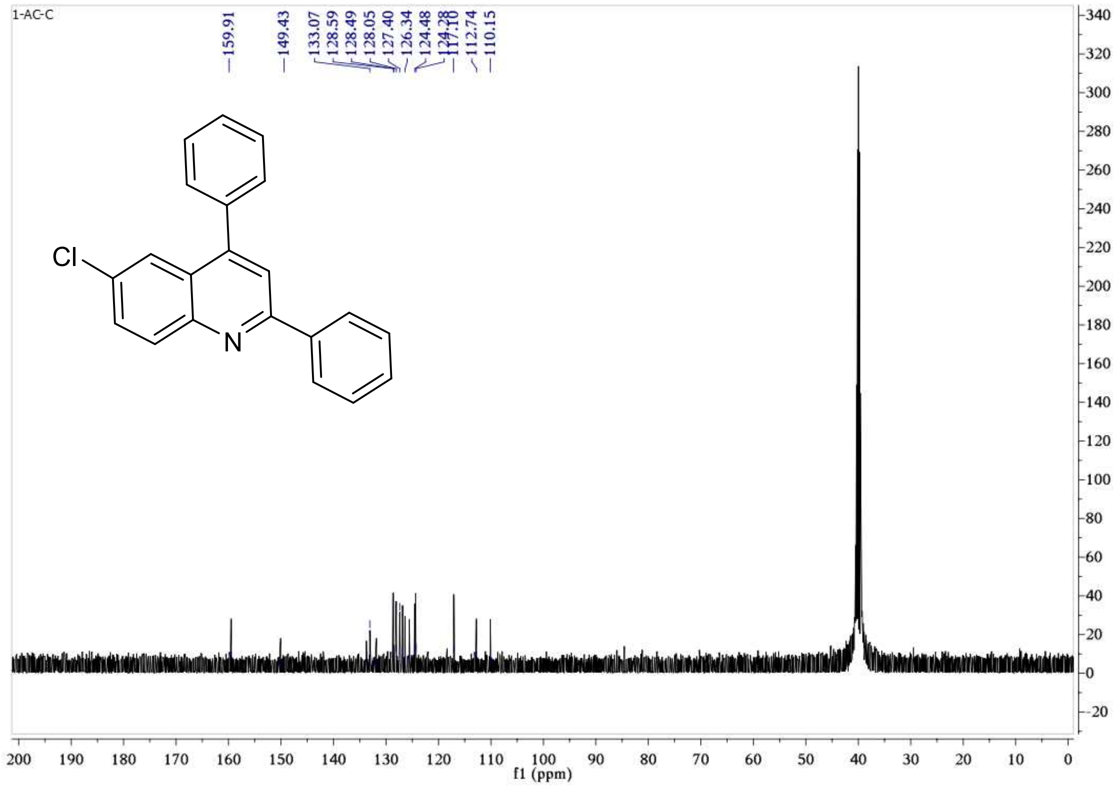

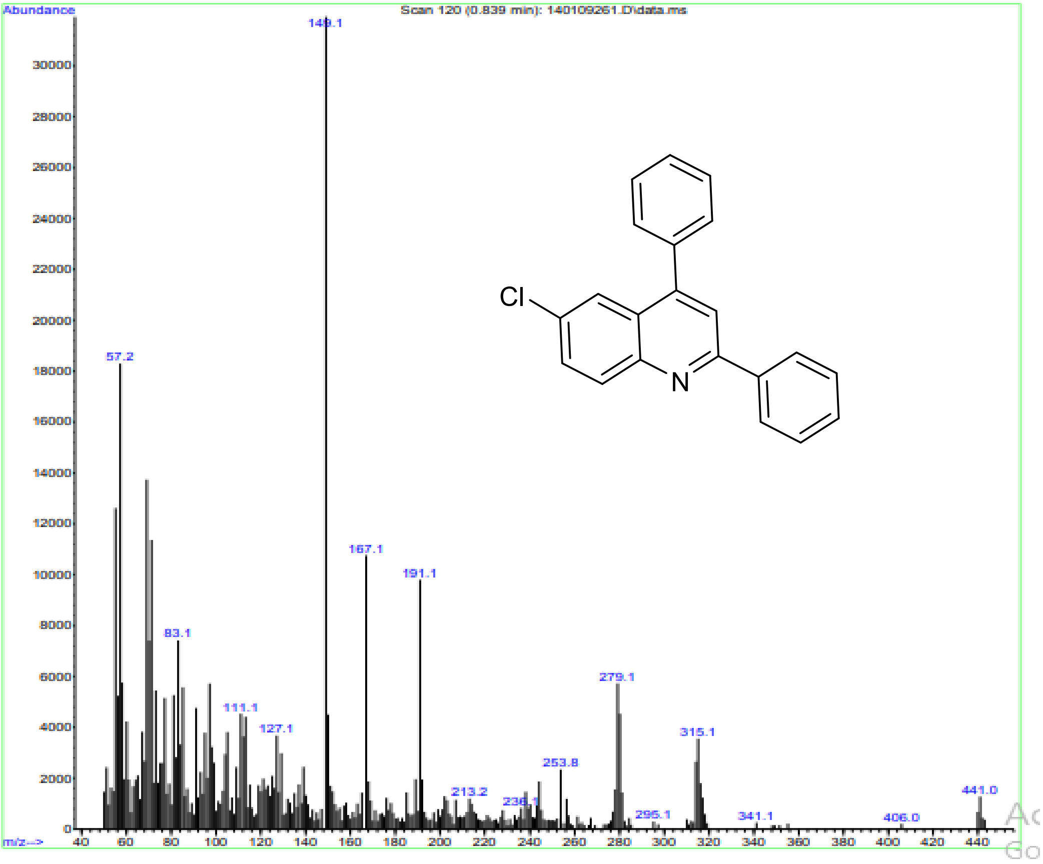

^{1}H NMR (500 MHz, DMSO-d_6, TMS) δ 8.84 (m, 3H), 8.16 (s, 1H), 8.09 (d, J = 7.6 Hz, 1H), 8.06 (s, 2H), 7.86 (s, 2H), 7.72 (d, J = 8.0 Hz, 1H), 7.66 (d, J = 2.5 Hz, 1H), 7.64 (d, J = 8.2 Hz, 1H), 7.53 (s, 1H). ^{13}C NMR (126 MHz, DMSO-d_6, TMS) δ 166.50, 164.13, 163.30, 151.38, 134.62, 133.79, 133.36, 133.13, 132.63, 131.91, 131.06, 130.74, 129.27, 128.34, 126.93, 126.02, 125.42, 120.81, MS m/z (%): 361.1.

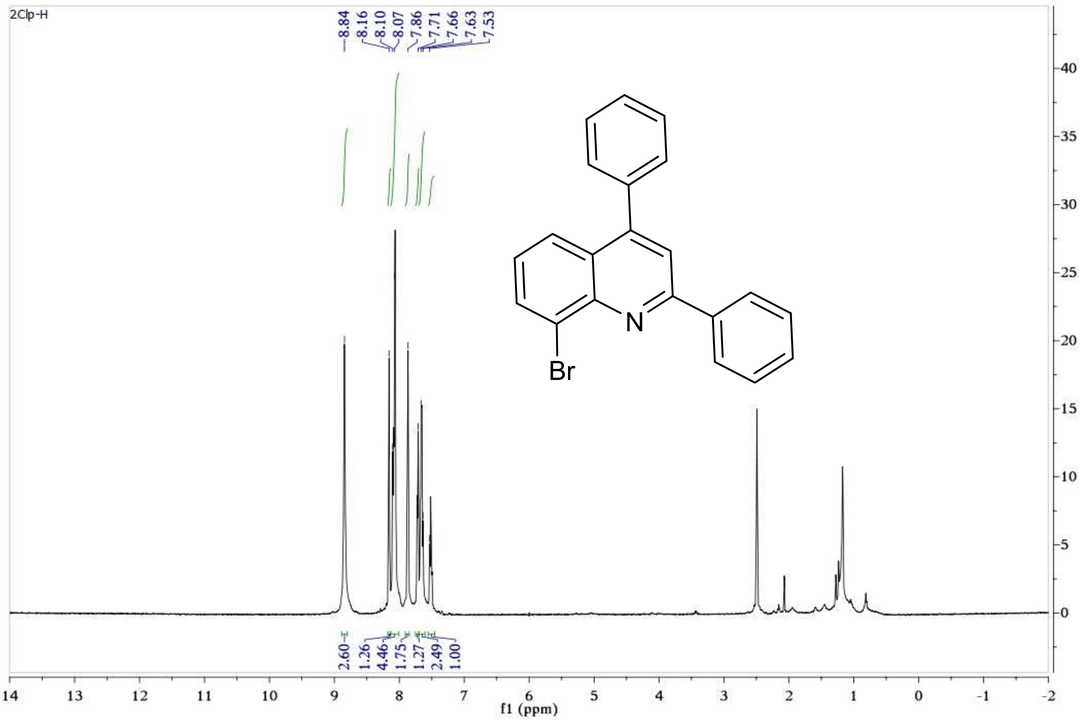
2Clp-H
8.84
8.16
8.10
8.07
7.86
7.71
7.66
7.63
7.53
N
Br
2.60
1.26
4.46
1.75
1.27
2.49
1.00
f1 (ppm)

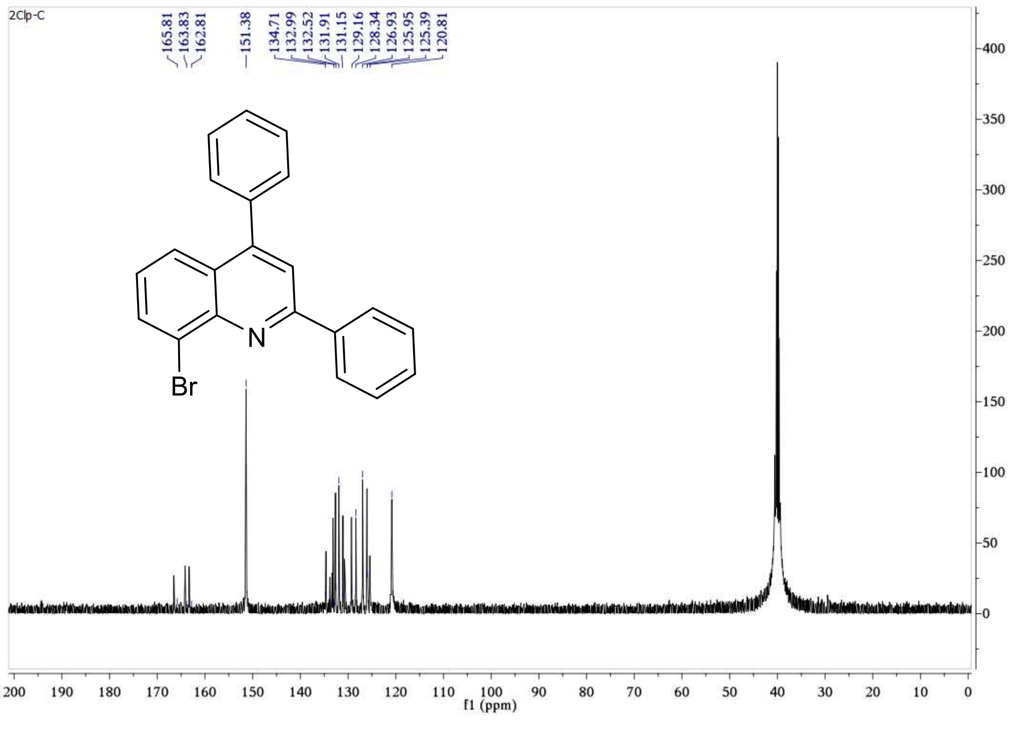
2Clp-C
165.81
163.83
162.81
151.38
134.71
132.99
132.52
131.91
131.15
129.16
128.34
126.93
125.95
125.39
120.81
N
Br
f1 (ppm)

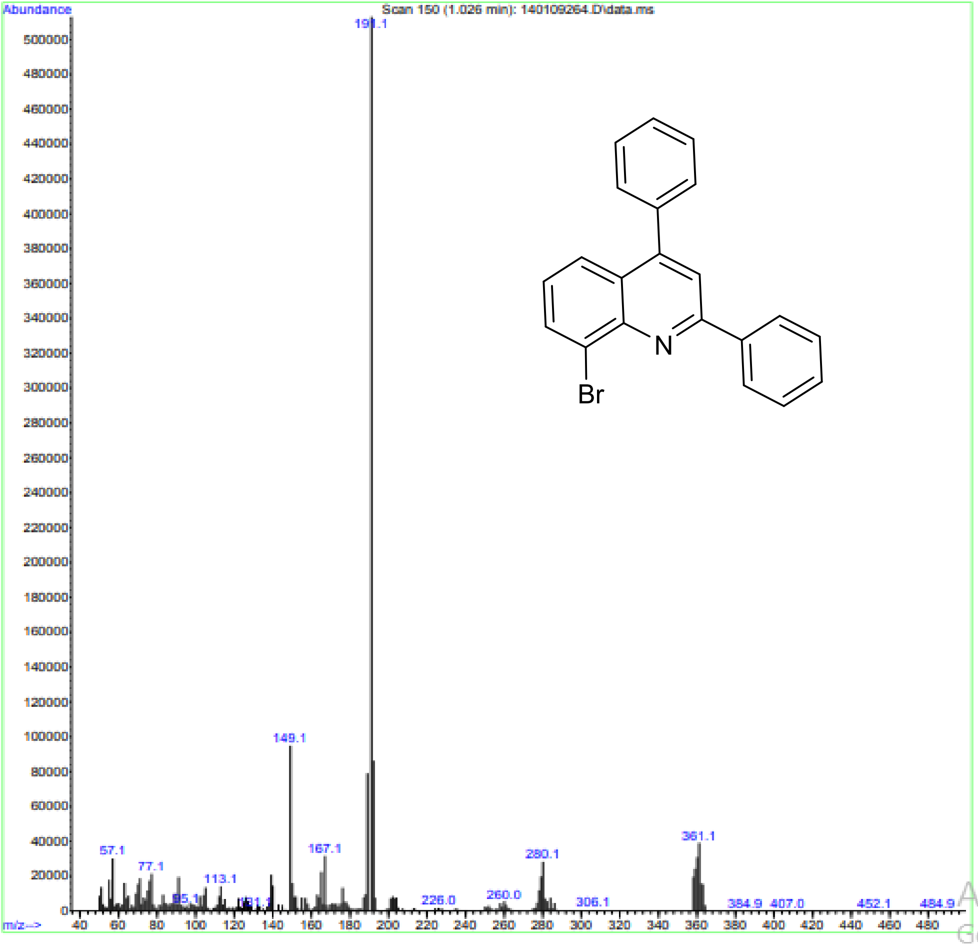

^{1}H NMR (500 MHz, DMSO-d_6, TMS) δ 8.89 – 8.85 (m, 3H), 8.85 (s, 1H), 8.11 (d, J = 6.7 Hz, 3H), 7.95 (d, J = 14.3 Hz, 2H), 7.61 (dd, J = 9.0, 4.0 Hz, 4H), 7.50 (s, 4H), 7.10 (d, J = 7.1 Hz, 1H), 3.85 (s, 3H). ^{13}C NMR (126 MHz, DMSO-d_6, TMS) δ 164.45, 160.22, 134.00, 132.41, 131.20, 129.85, 127.22, 123.82, 118.54, 117.51, 116.25, 112.65, 111.98, 56.13. MS m/z (%): 329.1.

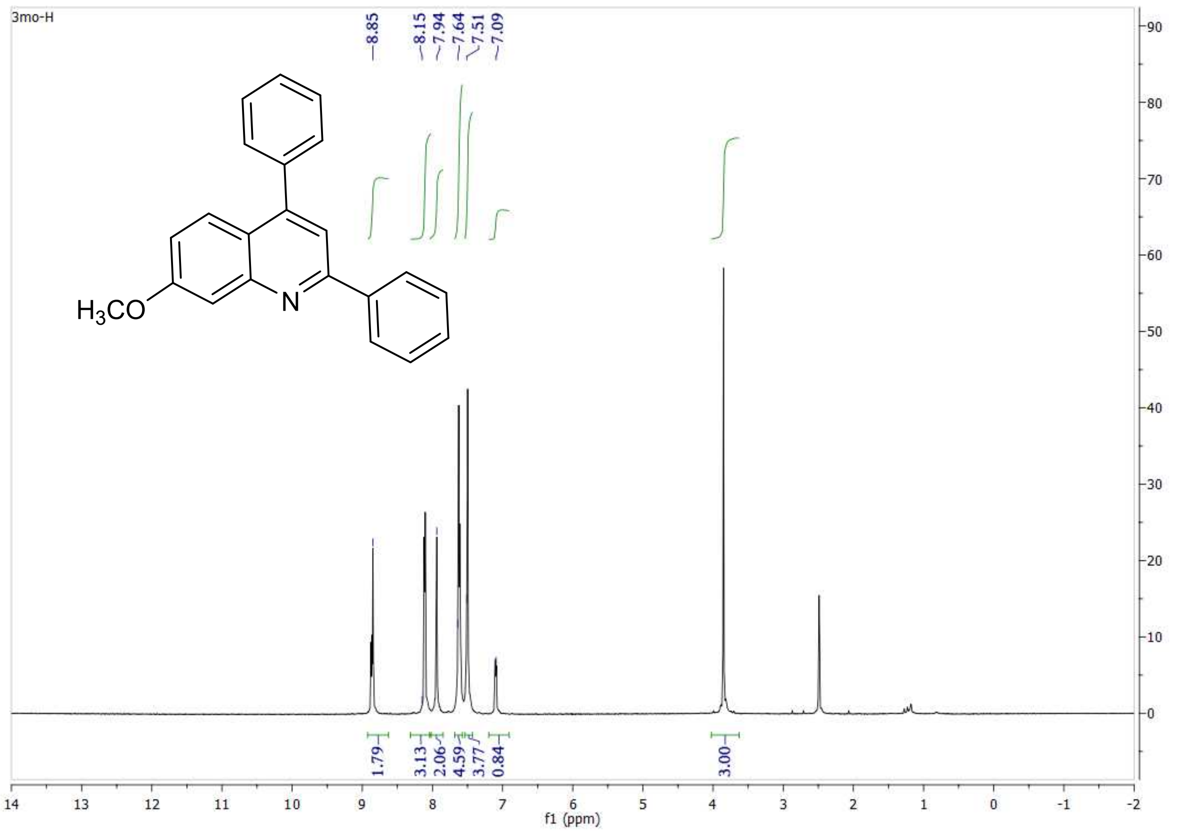
3mo-H
8.85
8.15
7.94
7.64
7.51
7.09
H3CO
N
1.79
3.13
2.06
4.59
3.77
0.84
3.00
f1 (ppm)

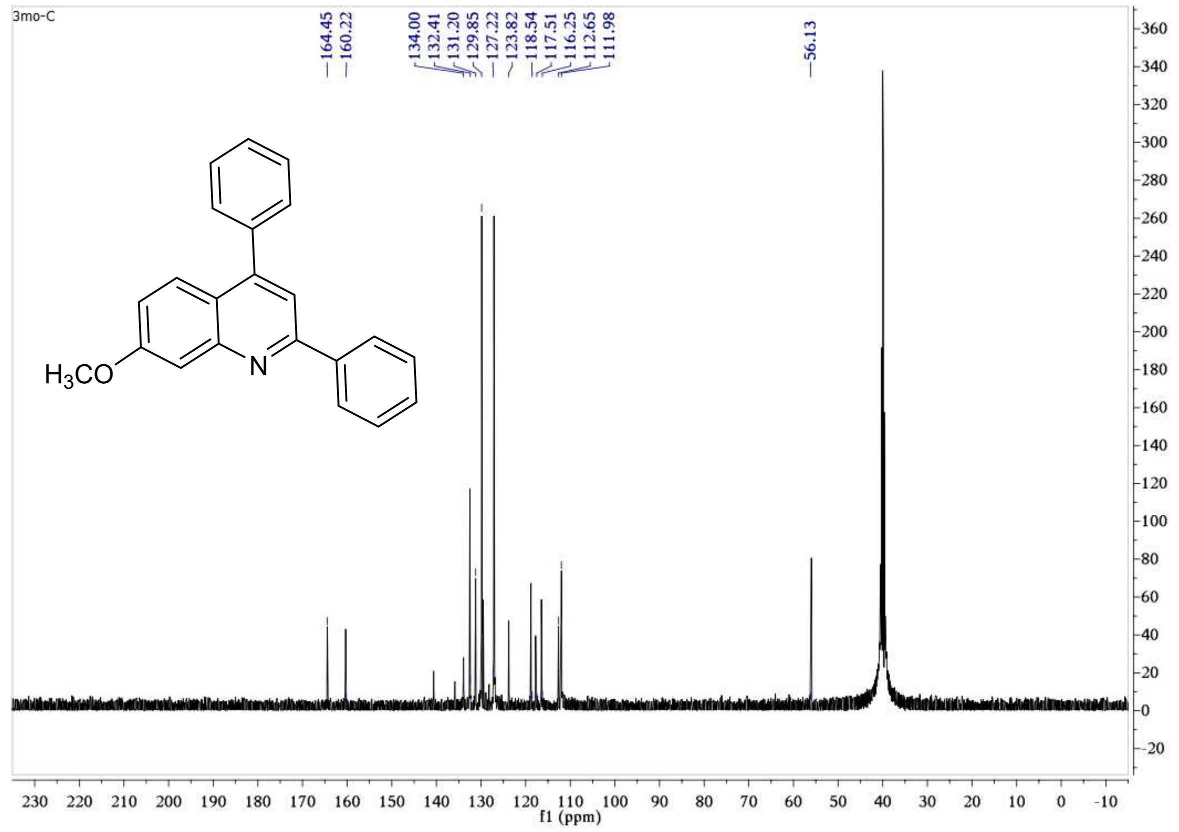
3mo-C
164.45
160.22
134.00
132.41
131.20
129.85
127.22
123.82
118.54
117.51
116.25
112.65
111.98
56.13
H3CO
N
f1 (ppm)

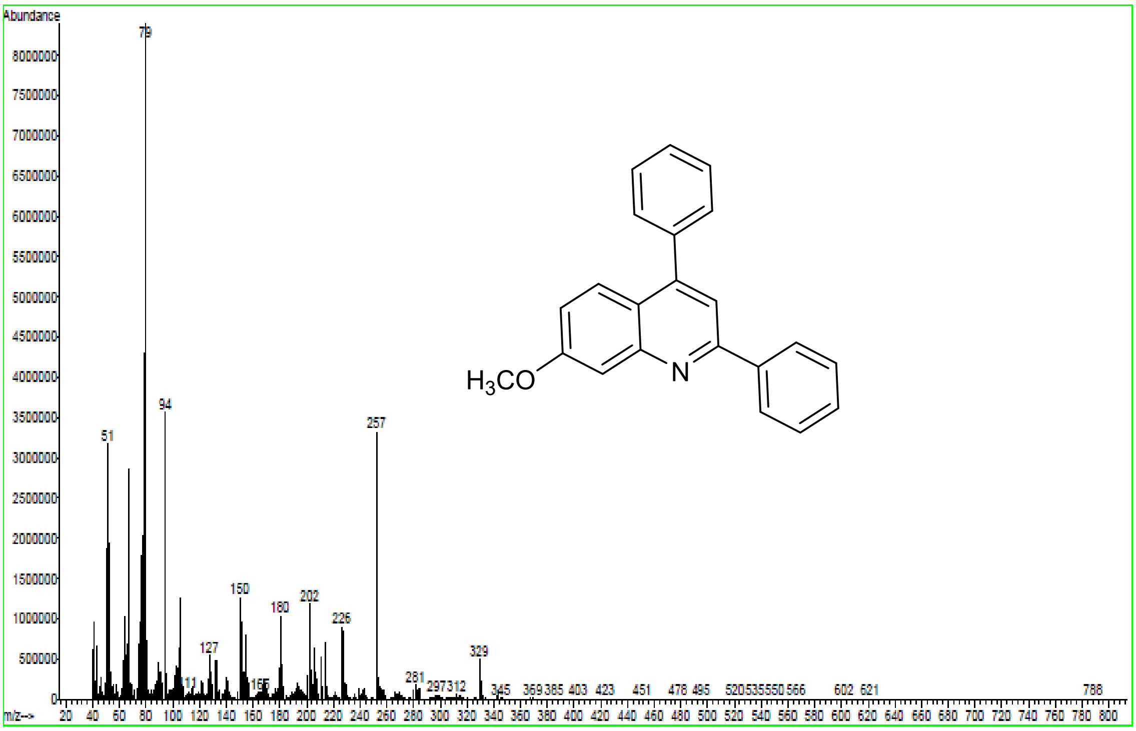

^{1}H NMR (500 MHz, DMSO-d_6, TMS) δ 8.27 (s, 1H), 8.13 (dd, J = 13.8, 7.7 Hz, 5H), 7.84 (d, J = 8.0 Hz, 2H), 7.62 (d, J = 7.4 Hz, 6H), 7.57 (s, 1H). ^{13}C NMR (126 MHz, DMSO-d_6, TMS) δ 165.18, 164.16, 163.52, 135.23, 132.72, 132.45, 132.04, 129.84, 129.48, 127.33, 127.13, 126.14, 125.87, 123.62, 122.96.

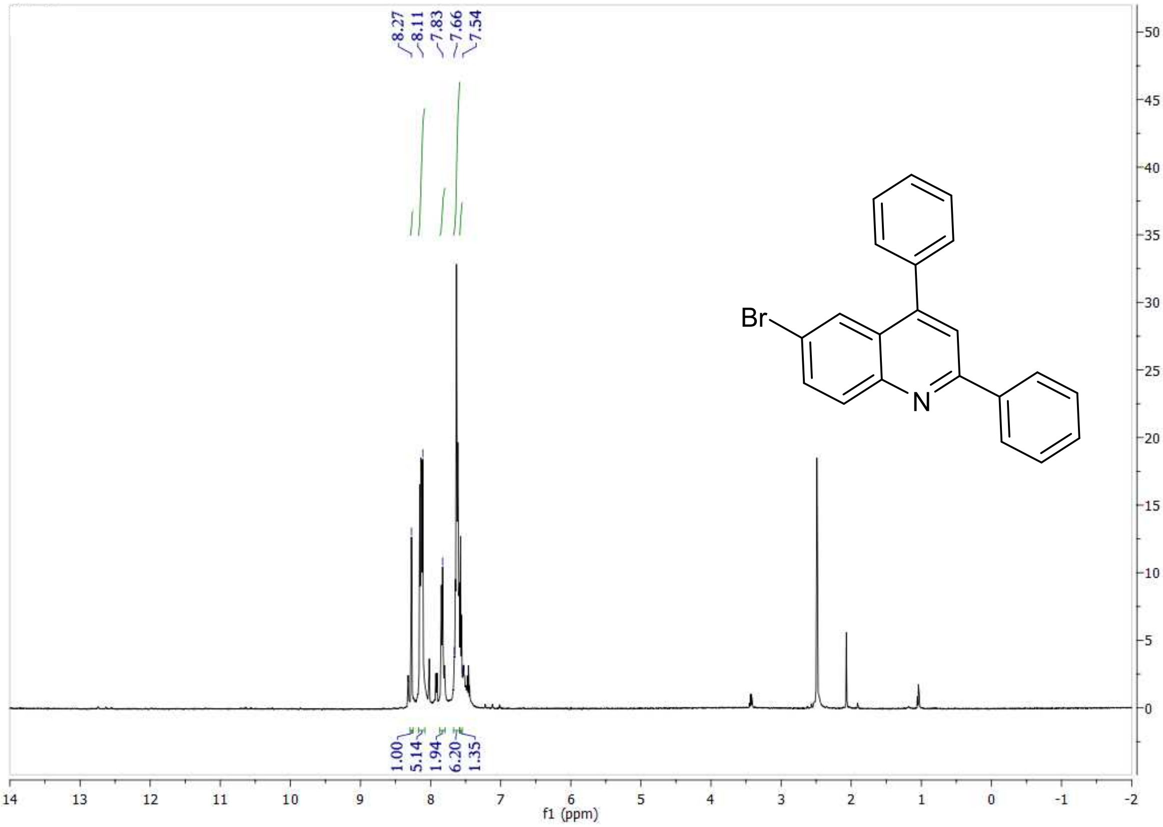

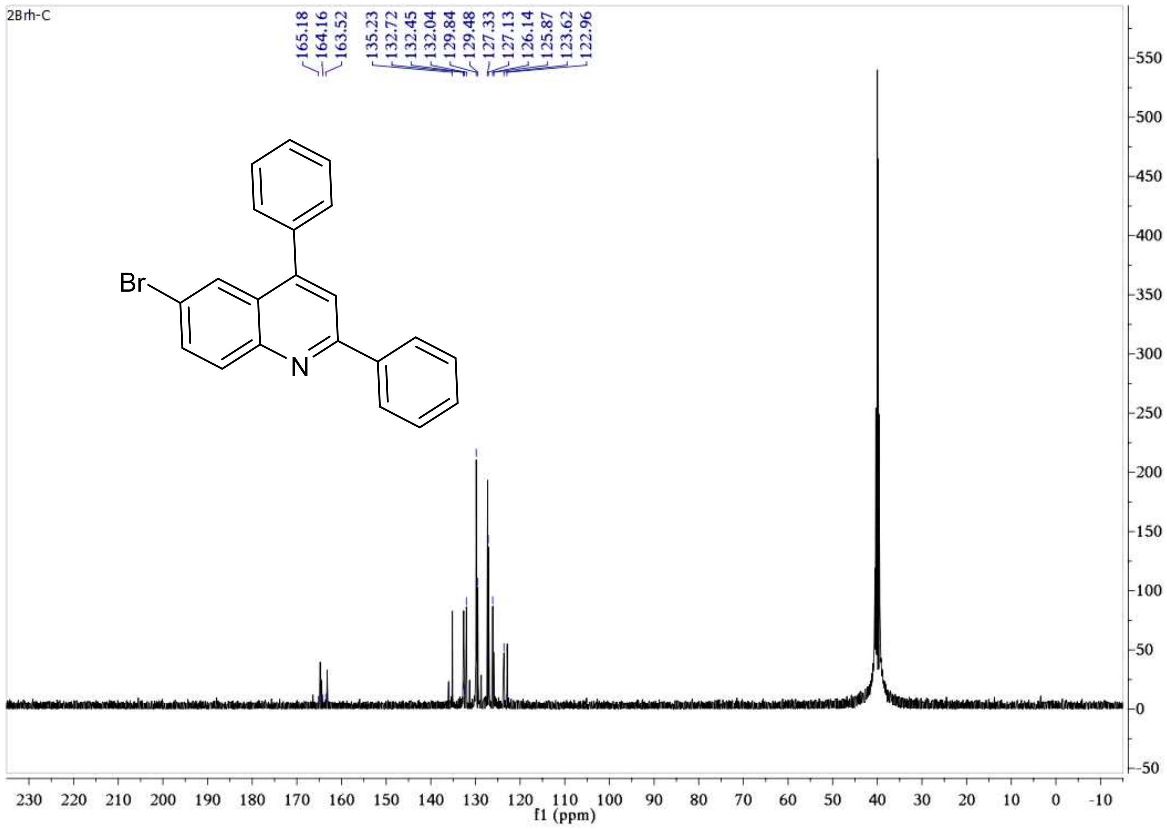

^{1}H NMR (500 MHz, DMSO-d_6, TMS) δ 8.78 (s, 1H), 8.64 (s, 1H), 8.56 (d, J = 6.4 Hz, 1H), 8.40 (d, J = 7.4 Hz, 1H), 8.20 – 8.11 (m, 2H), 7.74 (t, J = 11.2 Hz, 1H), 7.65 (s, 3H), 7.47 – 7.17 (m, 1H), 6.95 (s, 1H). ^{13}C NMR (126 MHz, DMSO-d_6, TMS) δ 161.32, 156.60, 149.43, 145.09, 142.17, 135.95, 132.01, 131.31, 128.08, 126.10, 122.17, 120.50, 116.88, 113.58, 111.28, MS m/z (%): 361.1.

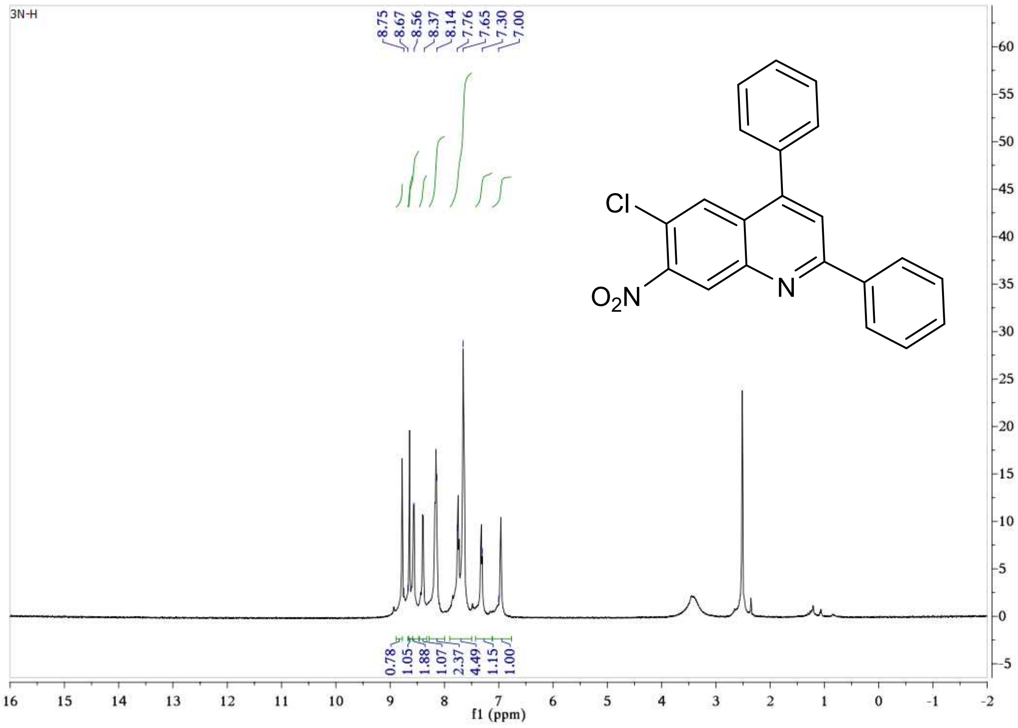

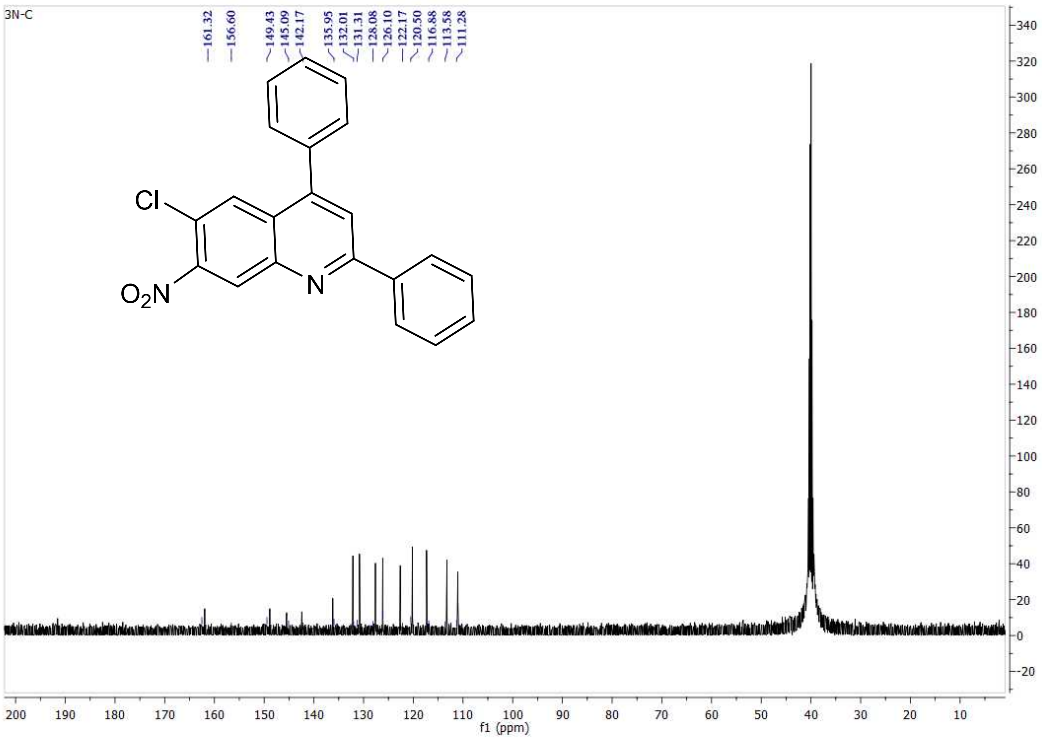
3N-C
161.32
156.60
149.43
145.09
142.17
135.95
132.01
131.31
128.08
126.10
122.17
120.50
116.88
113.58
111.28
Cl
O2N
N
f1 (ppm)

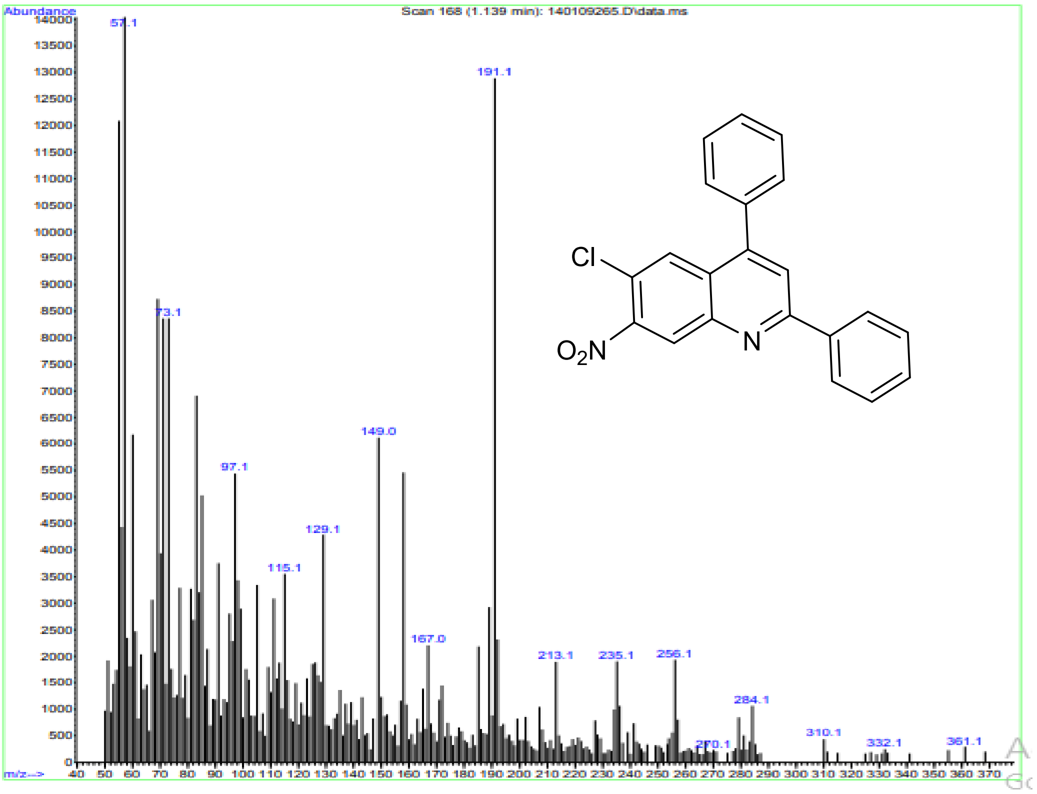
Abundance
Scan 168 (1.139 min): 140109265.D\data.ms
57.1
73.1
97.1
115.1
129.1
149.0
167.0
191.1
213.1
235.1
256.1
270.1
284.1
310.1
332.1
361.1
Cl
O2N
N
m/z-->

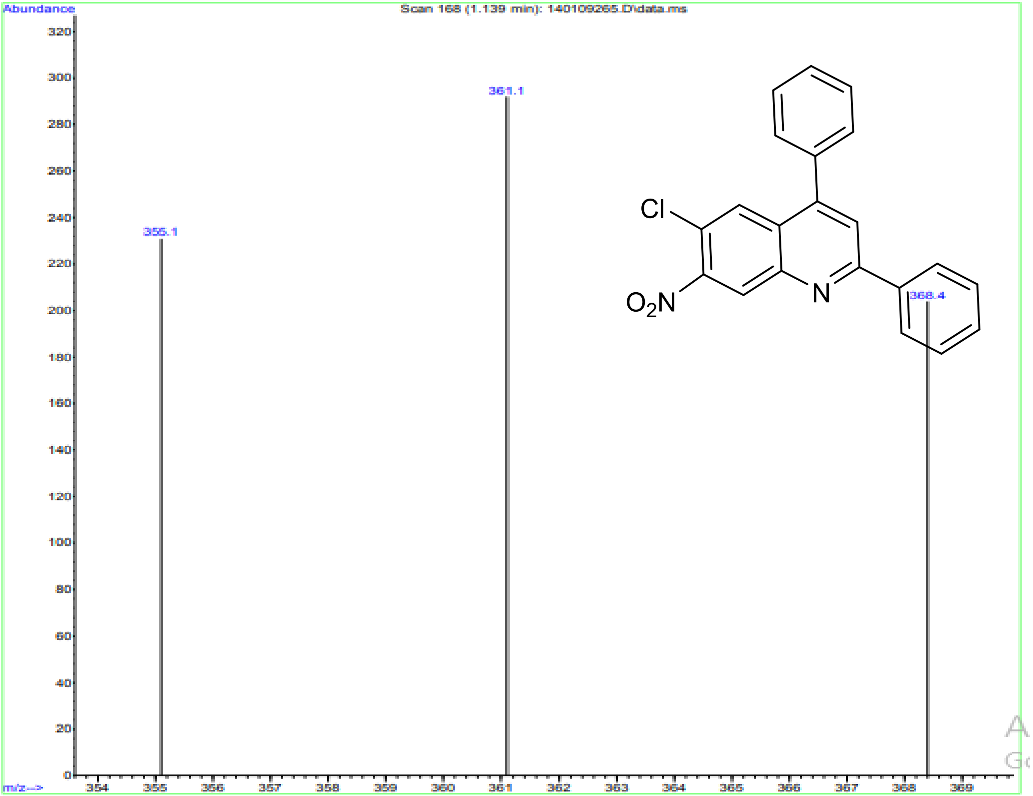

^{1}H NMR (400 MHz, DMSO-d_6, TMS) δ 8.90 (d, J = 2.1 Hz, 1H), 8.77 (dd, J = 4.8, 1.5 Hz, 1H), 8.19 – 8.12 (m, 2H), 7.83 – 7.75 (m, 4H), 7.63 (dd, J = 7.9, 4.9 Hz, 2H), 7.49 –7.40 (m, 4H), 7.03 (s, 2H), 6.96 (s, 2H), 2.60 (s, 3H). ^{13}C NMR (126 MHz, DMSO-d_6, TMS) δ 162.81, 155.06, 146.05, 145.23, 134.64, 131.88, 131.56, 131.38, 129.42, 129.15, 128.18, 125.71, 124.96, 23.35. MS m/z (%): 295.1.

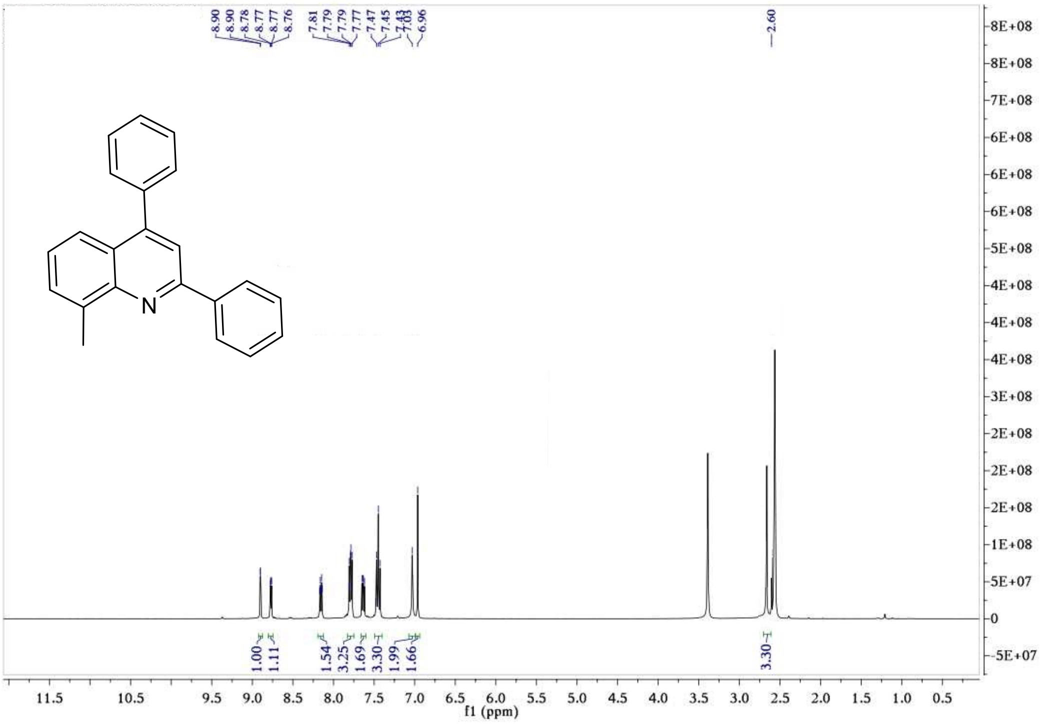

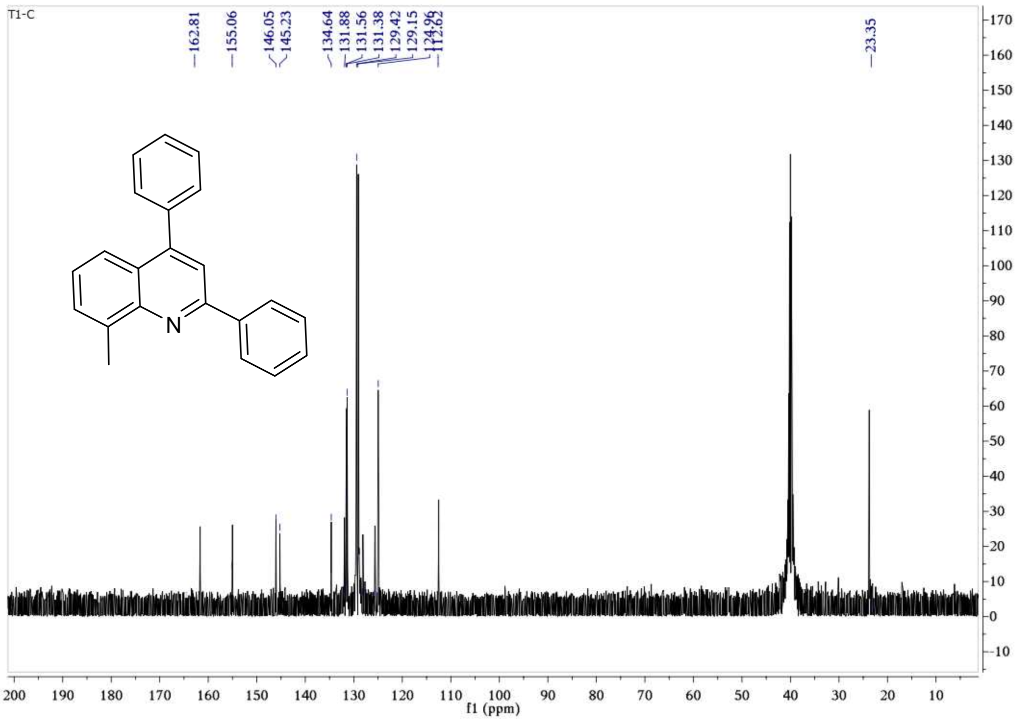
T1-C
162.81
155.06
146.05
145.23
134.64
131.88
131.56
131.38
129.42
129.15
124.96
112.62
23.35
N
f1 (ppm)

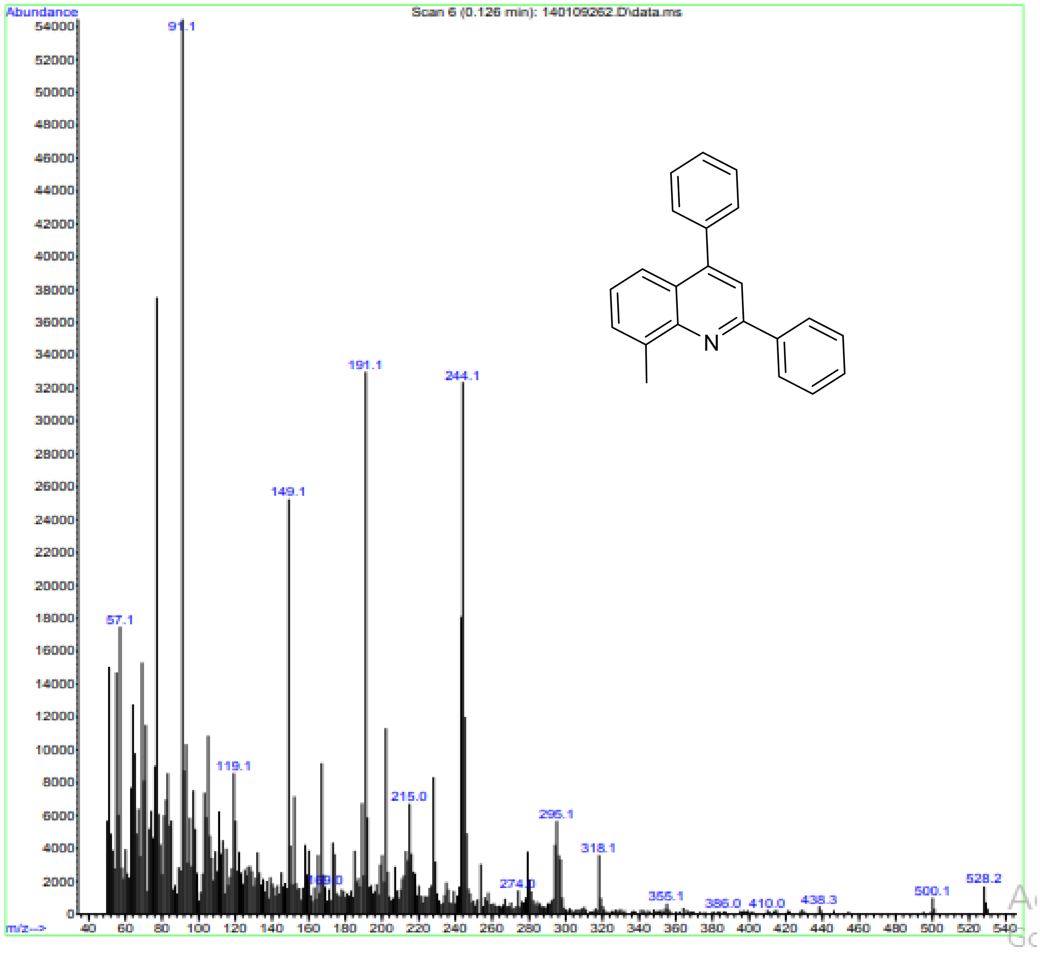
Abundance
Scan 6 (0.126 min): 140109262.D\data.ms
91.1
191.1
244.1
149.1
57.1
119.1
215.0
295.1
318.1
274.0
355.1
386.0
410.0
438.3
500.1
528.2
m/z-->
N

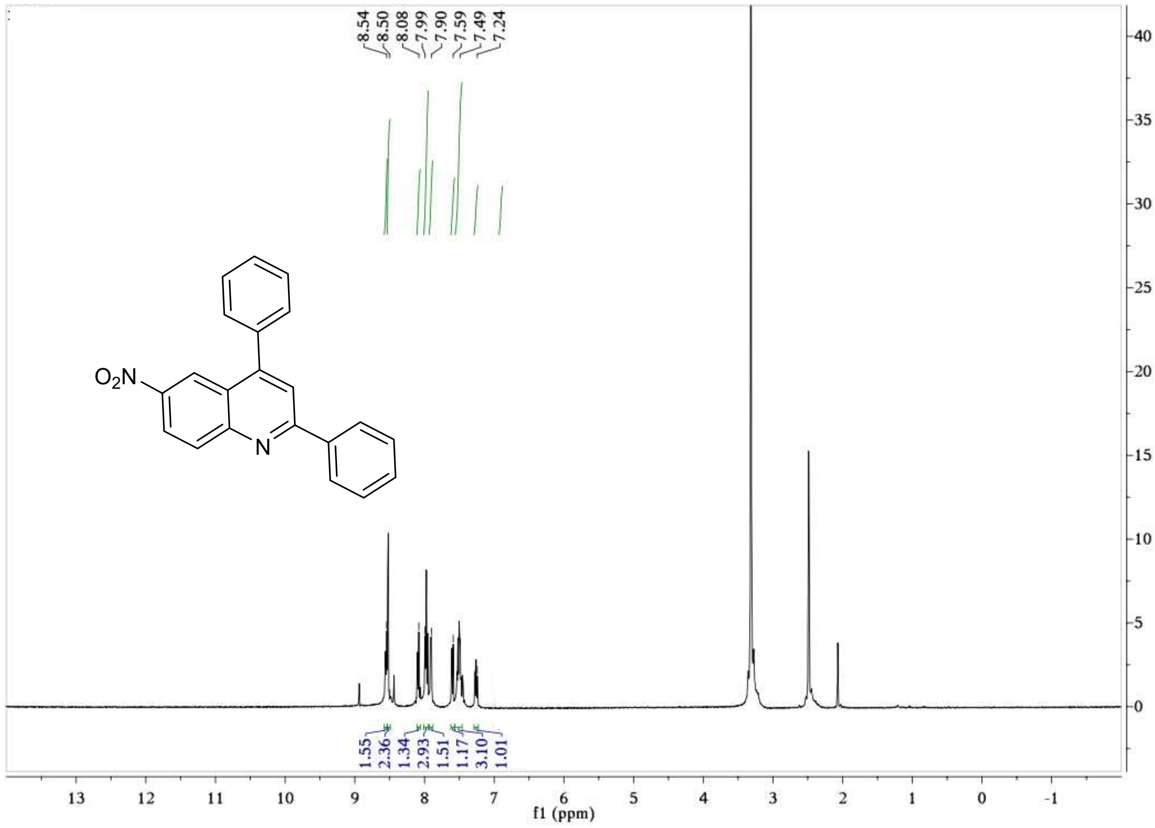

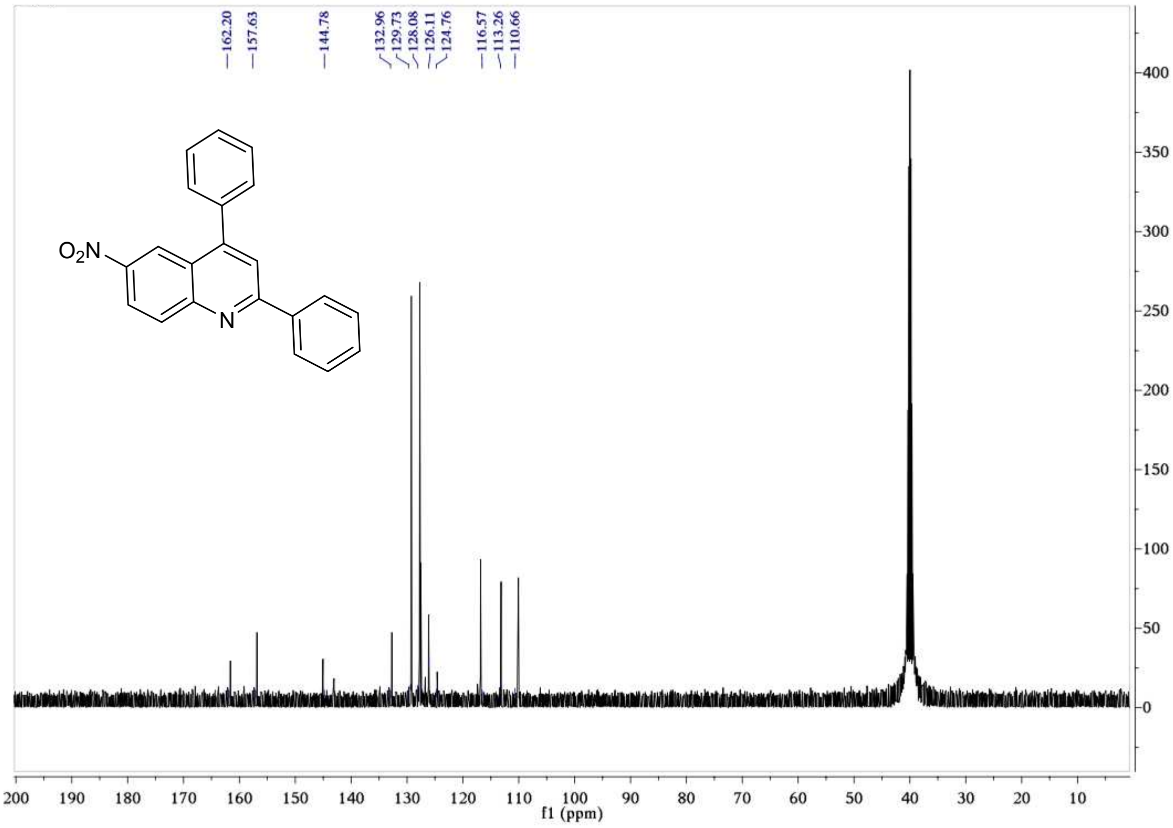

^{1}H NMR (500 MHz, DMSO-d_6) δ 8.49 (d, J = 6.8 Hz, 1H), 8.26 (s, 1H), 8.13 (d, J = 5 Hz, 2H), 7.69 – 7.58 (m, 2H), 7.59 – 7.44 (m, 3H), 7.23 (ddd, J = 8.8, 6.8, 1.4 Hz, 1H), 7.11 (dd, J = 5.1, 3.4 Hz, 1H),

6.88 (s, J = 6.8 Hz, 1H), 3.81 (s, 3H). ^{13}C NMR (126 MHz, dmso) δ 164.50, 148.22, 146.41, 132.62, 129.83, 127.04, 123.72, 121.83, 117.23, 116.52, 112.43, 111.73, 57.79.

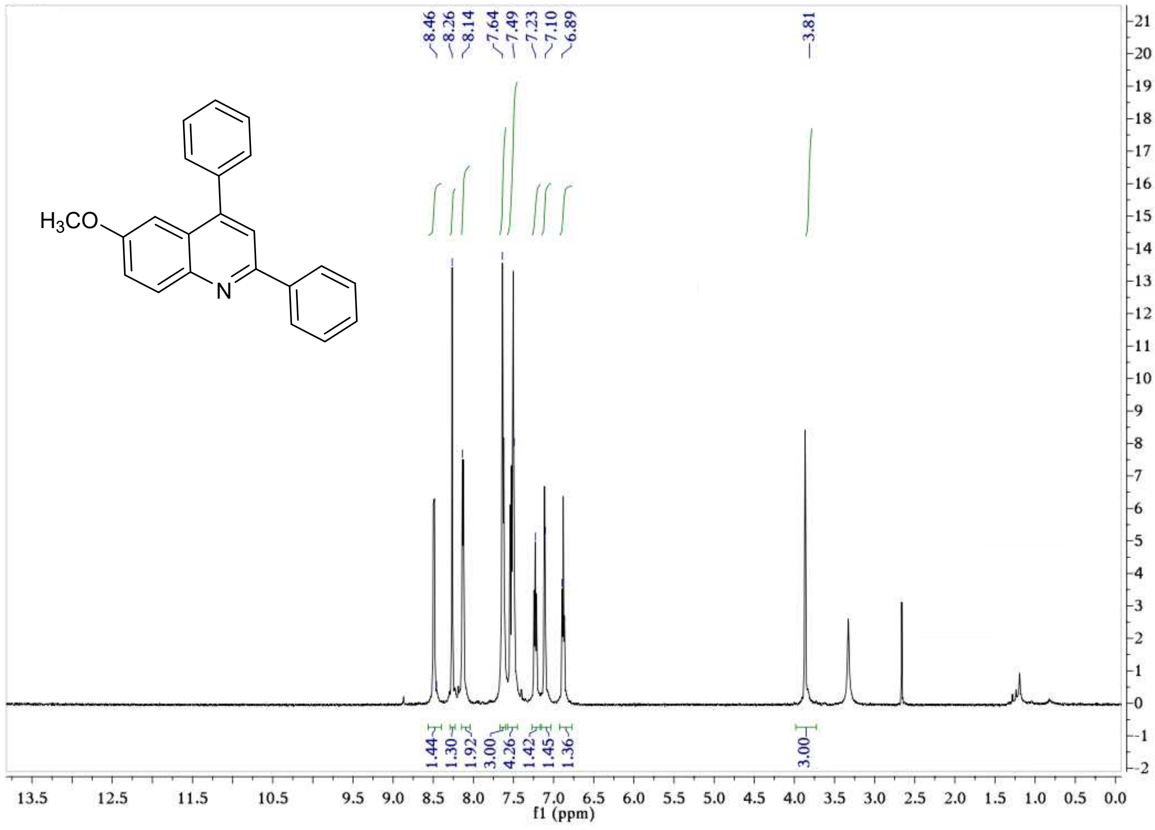

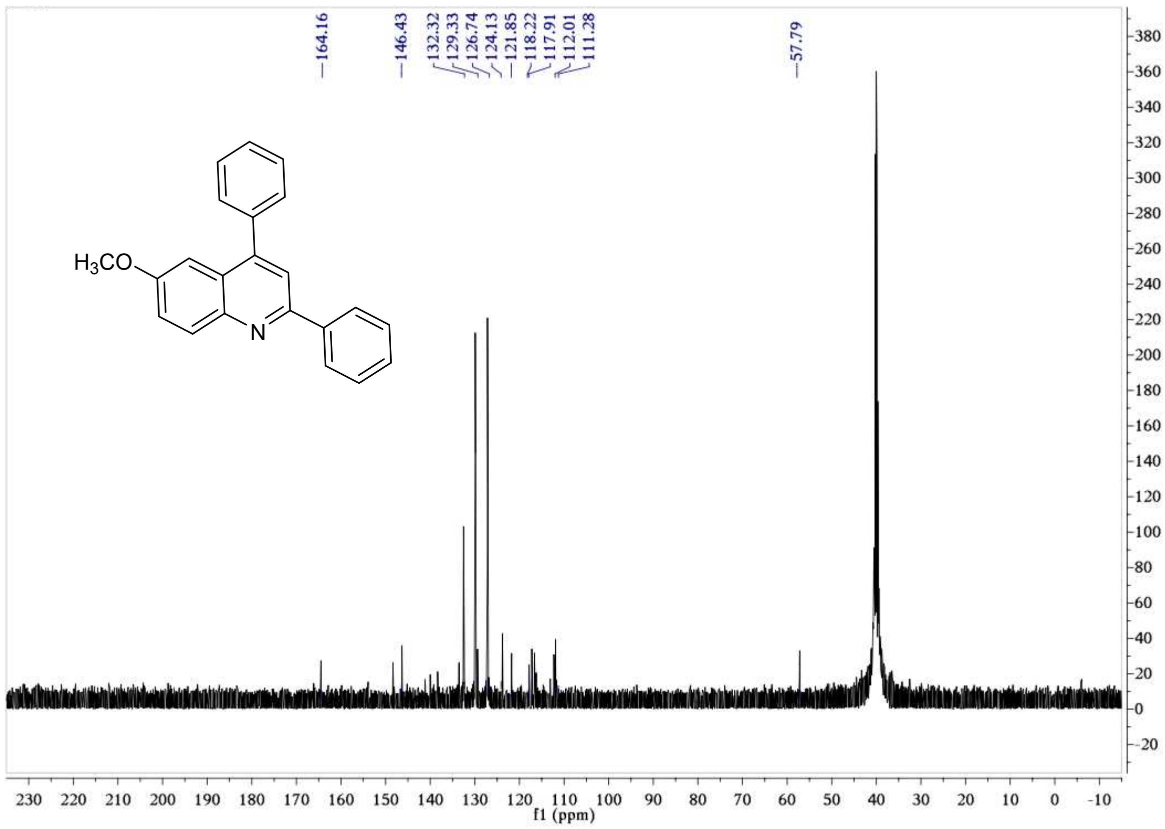

^{1}H NMR (500 MHz, DMSO-d_6) δ 8.93 – 8.81 (m, 2H), 8.13 (d, *J*= 10 Hz, 2H), 7.95 (d, *J* = 4.0 Hz, 1H), 7.92 – 7.80 (dd, *J* = 10, 12 Hz, 3H), 7.70– 7.60 (m, 4H), 7.50 (s, 1H). ^{13}C NMR (125 MHz, DMSO-d_6) δ 164.4, 140.9, 135.3, 133.8, 132.9, 132.5, 129.8, 129.5, 128.5, 127.1, 126.5, 124.1, 123.8, 117.6, 112.8, 112.1

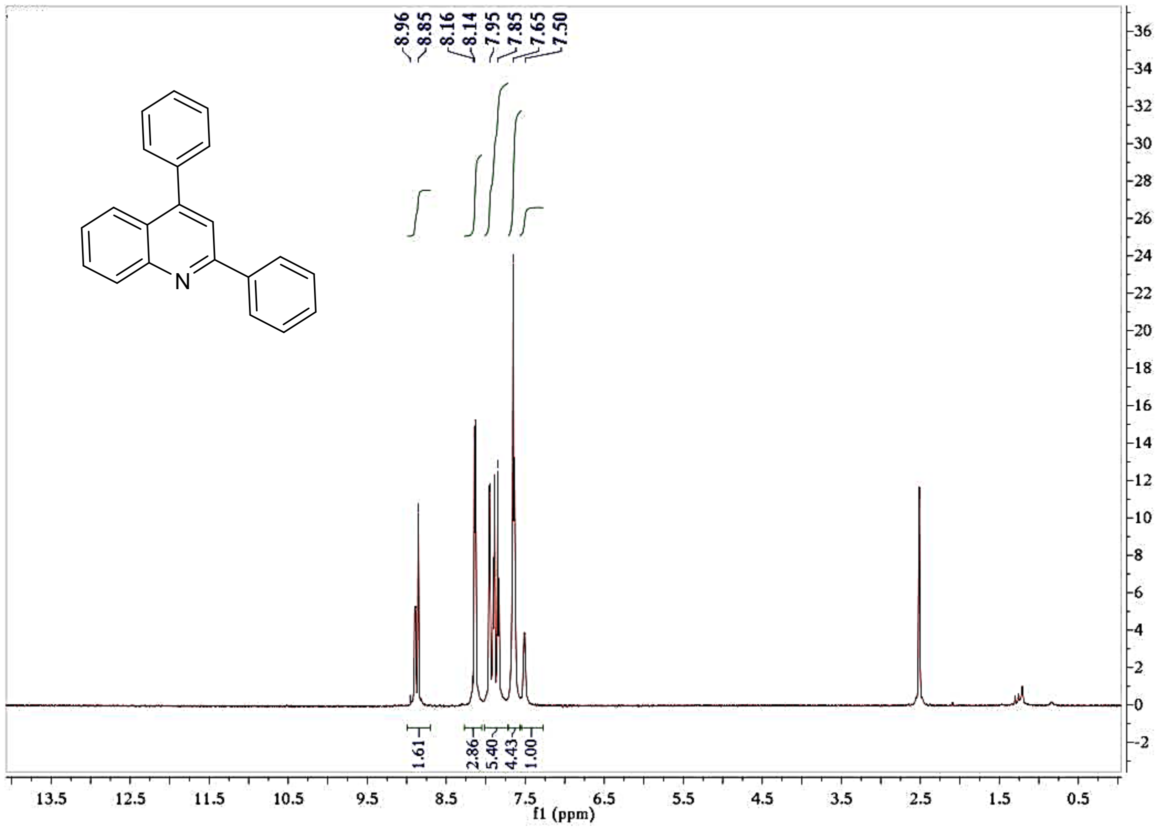

8.96
8.85
8.16
8.14
7.95
7.85
7.65
7.50
N
1.61
2.86
5.40
4.43
1.00
f1 (ppm)

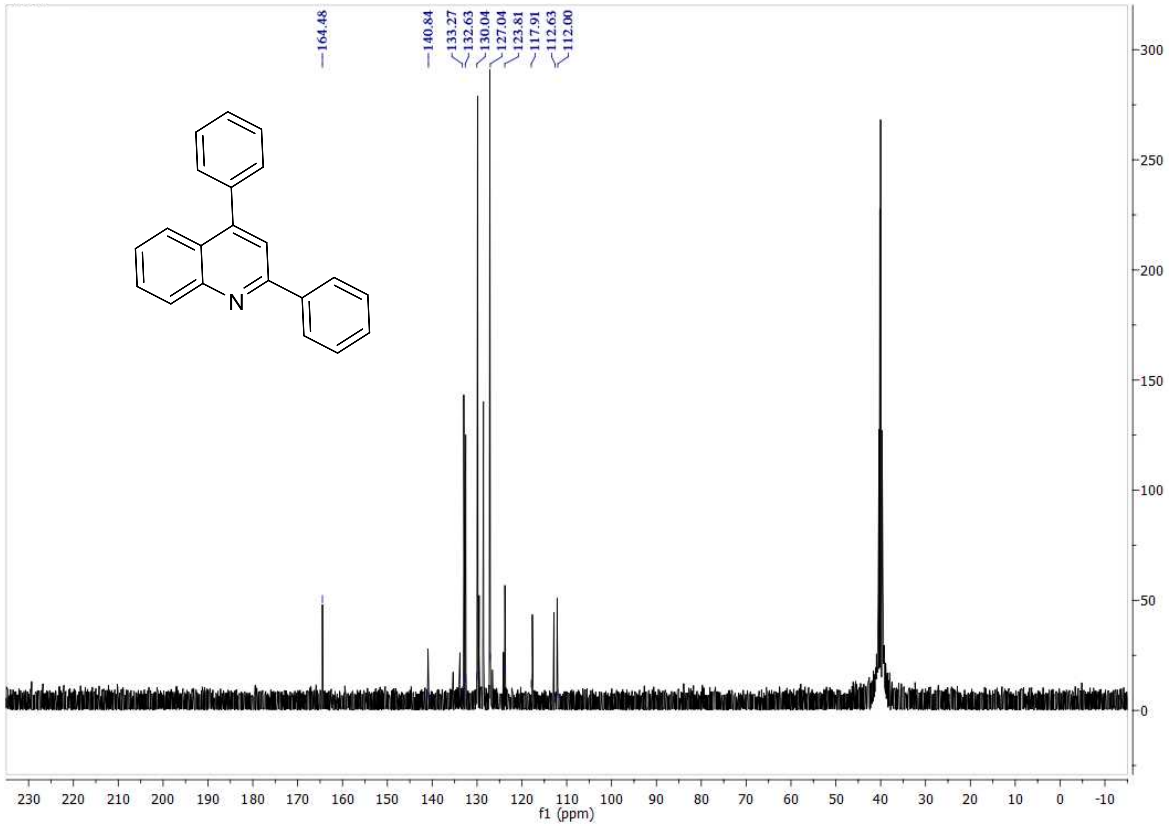

164.48
140.84
133.27
132.63
130.04
127.04
123.81
117.91
112.63
112.00
N
f1 (ppm)
